# Supplementary material for: Enhanced third-order optical nonlinearity in a dipolar carbene-metal-amide material with two-photon excited delayed fluorescence
Source: Commun Chem. 2026 Feb 17;9:135. doi: 10.1038/s42004-026-01928-5 (PMC13022332; doi:10.1038/s42004-026-01928-5)
Supplement: Supplementary file 2 — SUPPLEMENTAL MATERIAL [file 42004_2026_1928_MOESM2_ESM.pdf]

SUPPLEMENTARY INFORMATION

Enhanced Third-Order Optical Nonlinearity in a Dipolar Carbene-Metal-Amide Material with Two-Photon Excited Delayed Fluorescence

Ikechukwu D. Nwosu, Lujo Matasović, Tárcius N. Ramos, Nguyen Le Phuoc, Giacomo Londi, Alexander J. Gillett, Daniel Toolan, Charles T. Smith, George F. S. Whitehead, Mireille Blanchard-Desce, Jonathan Daniel, Mikko Linnolahti, Yoann Olivier, and Alexander S. Romanov

Supplementary Information Table of Contents

General Considerations 2

Experimental 2

Thermogravimetric Analysis 10

Single Crystal X-ray Crystallography 10

Cyclic Voltammetry 12

Photophysical Characterisation 13

2-Photon Absorption 16

Theoretical Calculations 26

Optimised coordinates 40

Supplementary References 51

Supplementary Methods.

General considerations. All reactions were performed under a N_2 atmosphere. Solvents were dried as required. 2,3-dichloroquinoxaline, 3,5-dimethylaniline, and triethyl orthoformate were purchased from commercial vendors and used as received. Carbazole was prepared according to the literature procedure. ^{1}H and $^{13}C\{^{1}H\}$ NMR spectra were recorded using a Bruker AVIII HD 400 MHz NMR spectrometer. ^{1}H NMR spectra (400 MHz) and ^{13}C NMR (400 MHz) were referenced to CD_2Cl_2 at δ 5.32 ppm (^{13}C, δ 53.84 ppm) or $CDCl_3$ at δ 7.26 ppm (^{13}C, δ 77.16 ppm). Elemental analyses were performed by the Microanalysis Laboratory at the University of Manchester. Mass spectrometry data were obtained by the Mass Spectrometry Laboratory at the University of Manchester. Thermogravimetric analysis was performed with a TA Instruments SDT650 simultaneous thermal analyzer under a stream of nitrogen.

Experimental.

Synthesis and Characterization

Note that reported synthetic routes for CMA complexes require the use of strong bases such as potassium hexamethyldisilazide (KHMDS) to obtain the key precursor – Carbene-Metal-Halide (**LMCl**, M = Cu, Ag or Au) complex. We developed a facile synthetic route towards **LMCl** that involves microwave heating (μW) of the ethoxy derivative (**LOEt**, Scheme 2) for 2 h in the presence of the corresponding metal halides to obtain copper and gold complexes in excellent yields.

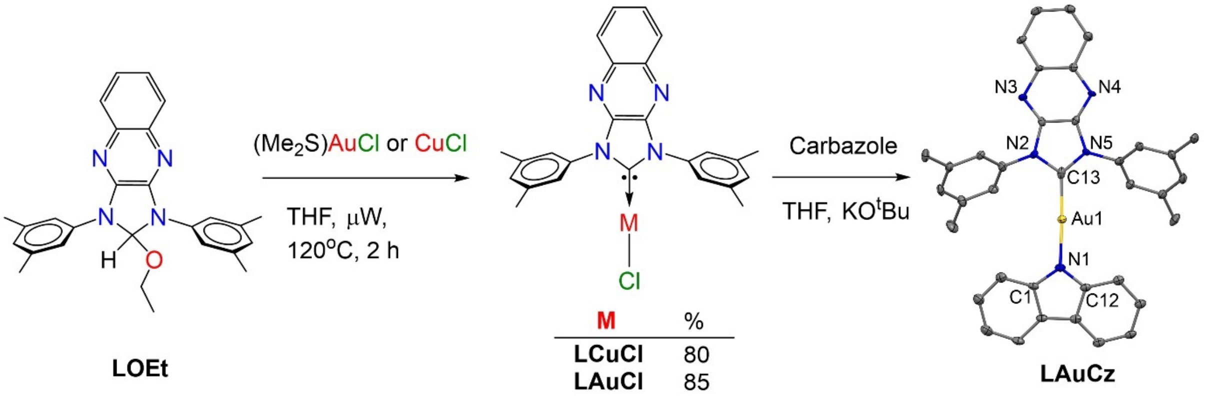

Scheme S1| Synthesis of the new CMA complex **LAuCz** and its crystal structure (right). Ellipsoids are shown at the 50% probability level. Hydrogen atoms are omitted for clarity.

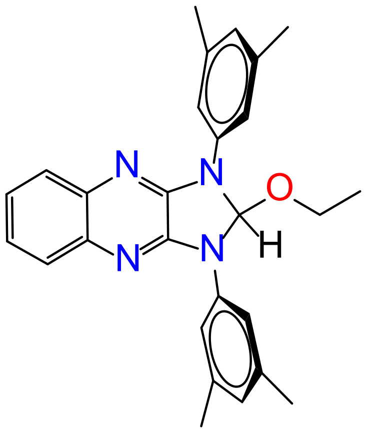

Synthesis of (QuinIm)OEt:

An oven-dried bomb flask equipped with a stir bar was charged with 2,3-dichloroquinoxaline (6 g, 0.03 mol) and 3,5-dimethylaniline (12 ml, 0.1 mol) under nitrogen. The solution mixture was stirred for 2 hours at 145 ºC, until a yellow solid formed. Triethyl orthoformate (30 ml, 0.18 mol) and concentrated HCl (3 ml) were added, and the solution was stirred overnight at 145ºC. Thereafter, the solution was left to cool slowly to room temperature, at which crystals were formed. The solution was filtered off and the crystals were washed with pentane and dried to give the desired product as off-white crystals. Yield 94% (12 g, 0.03 mol). ^{1}H NMR (400 MHz, CD_2Cl_2): δ 7.76 – 7.79 (m, 6H), 7.40 – 7.43 (m, 2H), 7.25 – 7.26 (d, 1H), 6.89 (s, 2H), 3.33 – 3.39 (q, 2H), 2.42 (s, 12H), 1.06 – 1.10 (t, 3H). $^{13}C\{^{1}H\}$ NMR (400 MHz, CD_2Cl_2): δ 143.7, 138.9, 137.4, 137.3, 126.7, 126.6, 125.6, 117.3, 96, 55.3, 21.8, 14.6.

^{1}H NMR (400 MHz, CDCl3)

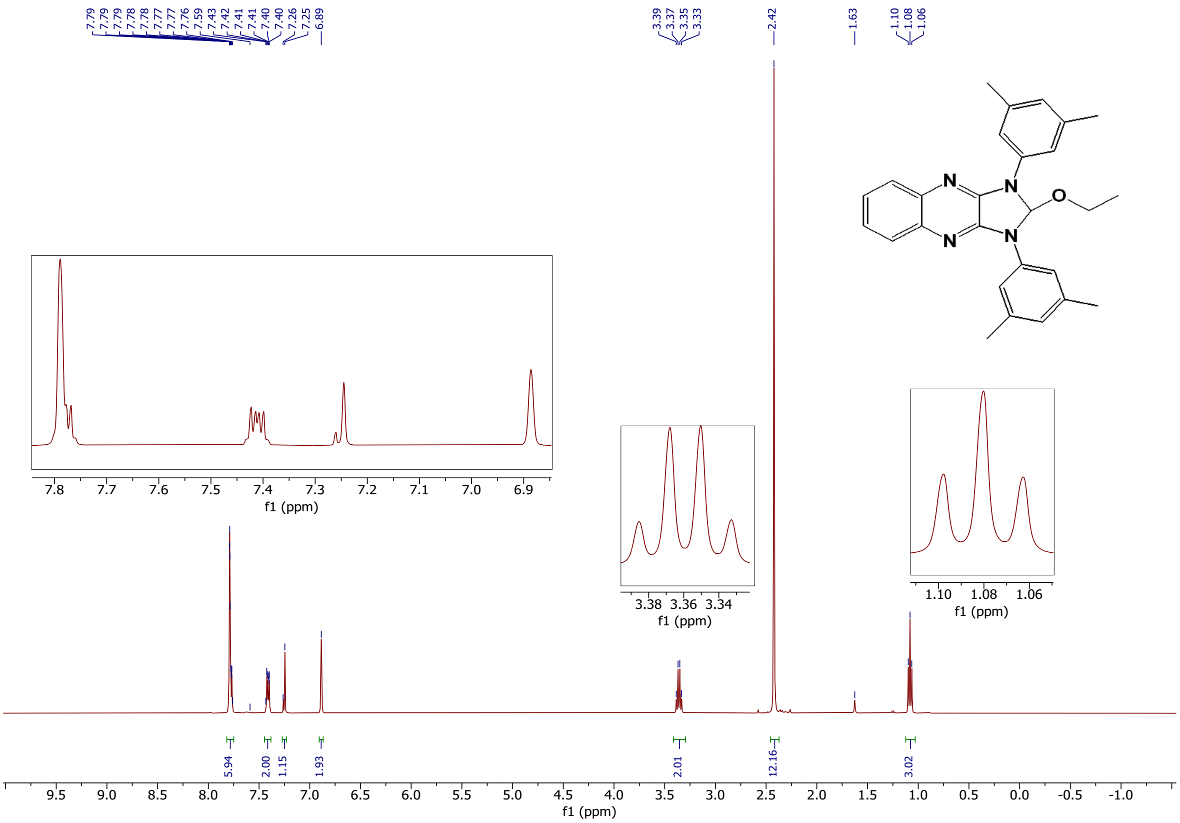

Figure S1. ^{1}H NMR (400 MHz, CD_2Cl_2)

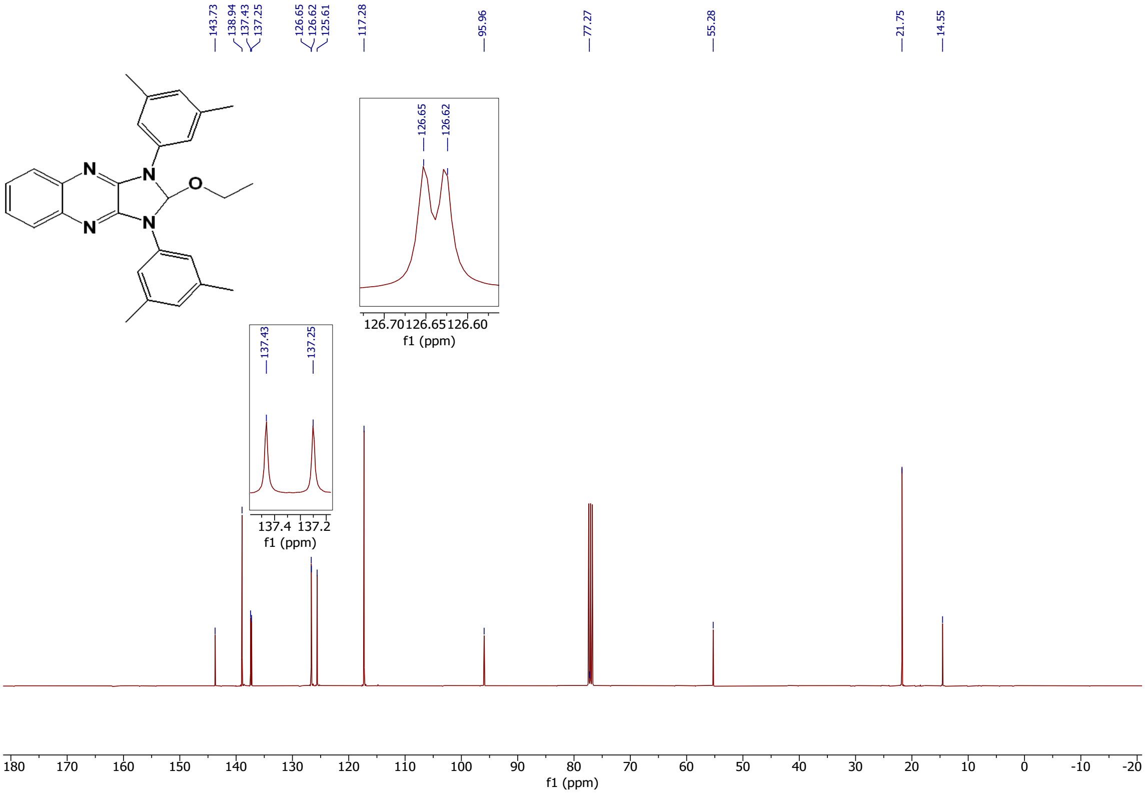

Figure S2. ^{13}C NMR (100 MHz, CD_2Cl_2)

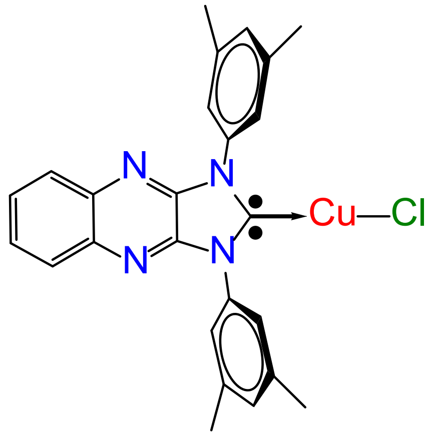

Synthesis of LCuCl:

Method 1: A solid mixture of (QuinIm)OEt (5 g, 0.01 mol) and CuCl (1.06 g, 10.7 mmol) was added dry THF (100 ml). The solution was stirred in refluxing THF for 5 hours, during which ethanol was completely evacuated via a Dean-Stark apparatus to promote the forward reaction. The reaction mixture was cooled to room temperature and volatiles were removed under vacuum. The crude solid was washed with THF (2 x 10 ml) and pentane (3 x 10 ml) and dried under vacuum to yield the pure product as a yellow solid in 70% yield (3.94 g, 8.25 mmol).

Method 2: A dry THF solution (40 ml) of L-ethoxy (2 g, 4.71 mmol) and CuCl (424 mg, 4.28 mmol) was heated at 120°C in a microwave synthesizer for 1 hour. The reaction mixture was cooled to room temperature and evaporated under vacuum. The resulting solid was washed with THF (2 x 10 ml) and pentane (3 x 10 ml) and dried under vacuum to yield the pure product as a yellow solid in 80% yield (1.8 g, 3.77 mmol). ^{1}H NMR (400 MHz, CD_2Cl_2): δ 8.26 - 8.21 (m, 2H), 7.91 – 7.87 (m, 2H), 7.55 (s, 4H), 7.31 (s, 2H), 2.51 (s, 12H). HRMS $C_{25}H_{22}ClCuN_4$ theoretical $[M+H]^+$ = 476.0824, HRMS (APCI(ASAP): = 476.0834

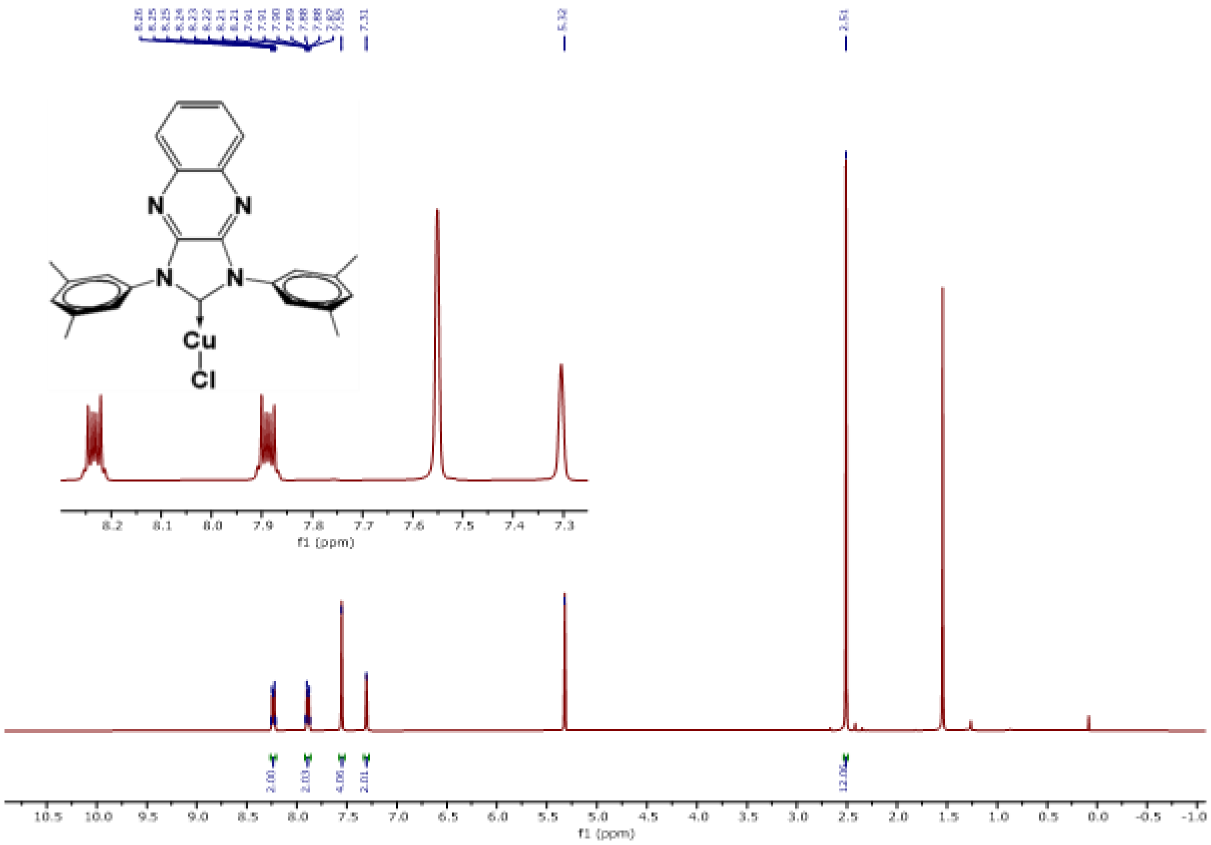

Figure S3. ^{1}H NMR (400 MHz, CD_2Cl_2)

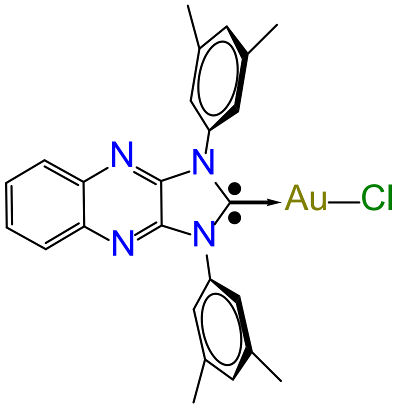

Synthesis of LAuCl:

Method 1: To a solid mixture of LCuCl (1.08 g, 2.27 mmol) and chlorodimethylsulphide gold (I) (0.67 g, 2.27 mmol, 1 eq.) was added dry CH_2Cl_2 (80 ml). The solution was sonicated for 10 minutes and stirred overnight at room temperature. Volatiles were removed under reduced pressure. The product was extracted with CH_2Cl_2, filtered through silica gel, and concentrated on a rotary evaporator. The product was precipitated with hexane, centrifuged and the

supernatant decanted to give a white solid, which was washed with hexane and pentane successively and dried to give the pure product as a white powder in 85% yield (1.18 g, 1.93 mmol).

Method 2: A dry THF (60 ml) solution of (QuinIm)OEt (2.98 g, 7.02 mmol) and chlorodimethylsulphide gold (I) (2.05 g, 6.94 mmol) was heated at 120ºC in a microwave synthesizer for 2 hours. The reaction mixture was cooled to room temperature and evaporated under vacuum. The product was extracted with DCM, filtered through silica gel, and concentrated on a rotary evaporator. The product was precipitated with hexane, centrifuged and the supernatant decanted to give a white solid, which was washed successively with hexane and pentane and dried to give the pure product as a white powder in 80% yield (3.43 g, 5.62 mmol). ^{1}H NMR (400 MHz, CD_2Cl_2): δ 8.23 - 8.19 (m, 2H), 7.91 – 7.87 (m, 2H), 7.45 (s, 4H), 7.35 (s, 2H), 2.51 (s, 12H).

^{1}H NMR (400 MHz, CD_2Cl_2)

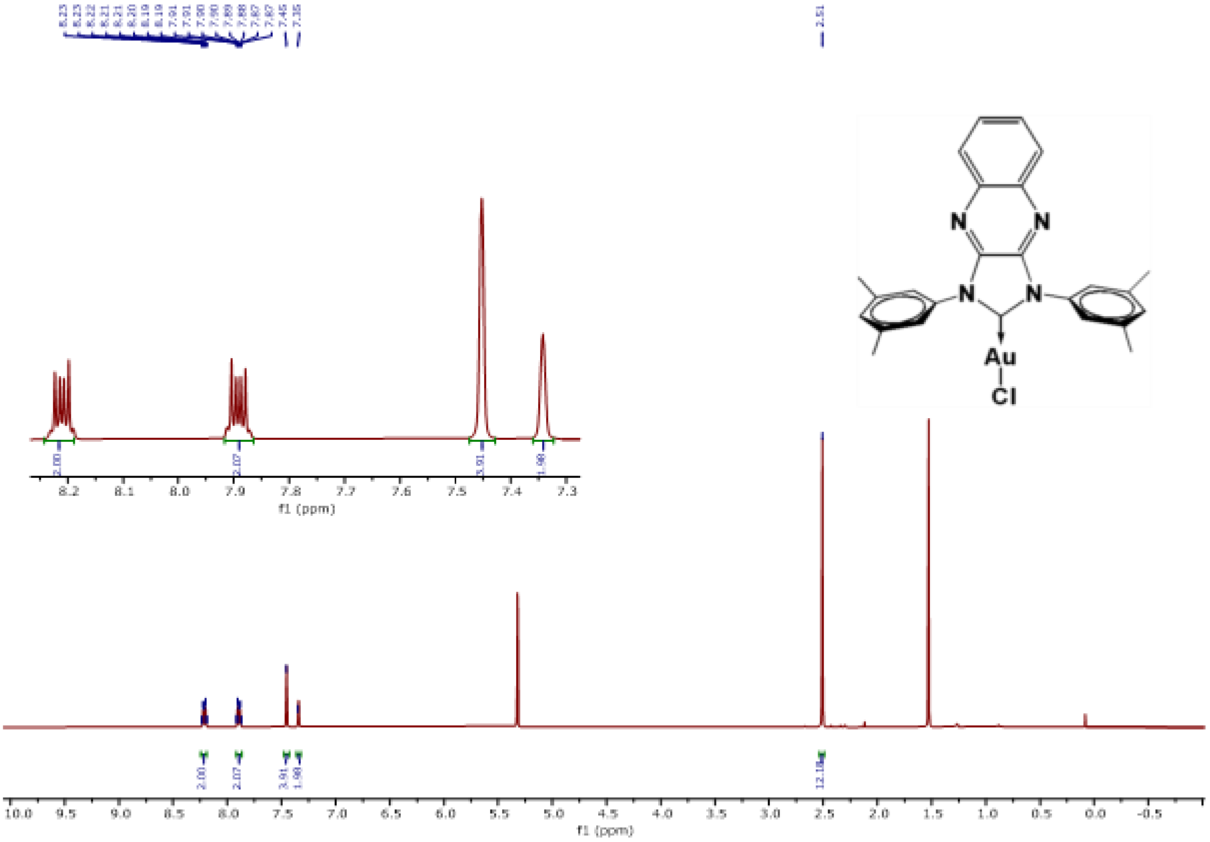

Figure S4. ^{13}C NMR (CD_2Cl_2)

Synthesis of LAuCz:

An oven dried Schlenk flask was charged with (QuinoxNHC)AuCl (500 mg, 0.82 mmol), carbazole (150.5 mg, 0.9 mmol) and KO^{t}Bu (121 mg, 1.08 mmol), followed by addition of THF (60 ml) under nitrogen. The solution was stirred overnight at room temperature. Afterwards, all volatiles were removed under reduced pressure. The residue was washed with copious amounts of diethyl ether, re-dissolved in CH_2Cl_2, filtered through a 2 cm celite layer, and evaporated to dryness. The resulting solid residue was washed with small amounts of diethyl ether to give orange solids as product. Yield 75% (455 mg, 0.61 mmol). Complex **LAuCz** has a good solubility in polar organic solvents (CH_2Cl_2, 1,2,-difluorobenzene and THF) and average solubility is toluene or chlorobenzene whereas it's poorly soluble in hexane or pentane.

^{1}H NMR (400 MHz, CD_2Cl_2): δ 8.23 – 8.28 (m, 2H,2 x *o*-CH Quinox), 7.97 – 7.99 (d, 2H, 2 x CH Cz), 7.89 – 7.93 (m, 2H, 2 x *m*-CH Quinox), 7.69 (s, 4H, 4 x *o*-CH dimethylPhen), 7.47 (s, 2H, 2 x *p*-CH dimethylPhen), 7.13 – 7.22 (m, 4H, 4 x CH Cz), 6.96 – 7.00 (m, 2H 2 x CH Cz), 2.60 (s, 12H, 4 x CH_3 dimethylPhen).

^{13}C NMR (400 MHz, CD_2Cl_2): δ 193.3 (C:), 149.3 (NCN Quinox-C-Im), 140.4 (CNAu, Cz), 140.2, 139.8, 135.6, 131.9, 130.2, 129.1, 124.9, 124, 123.7, 119.4, 116.5, 113.5, 21.3 (CH_3).

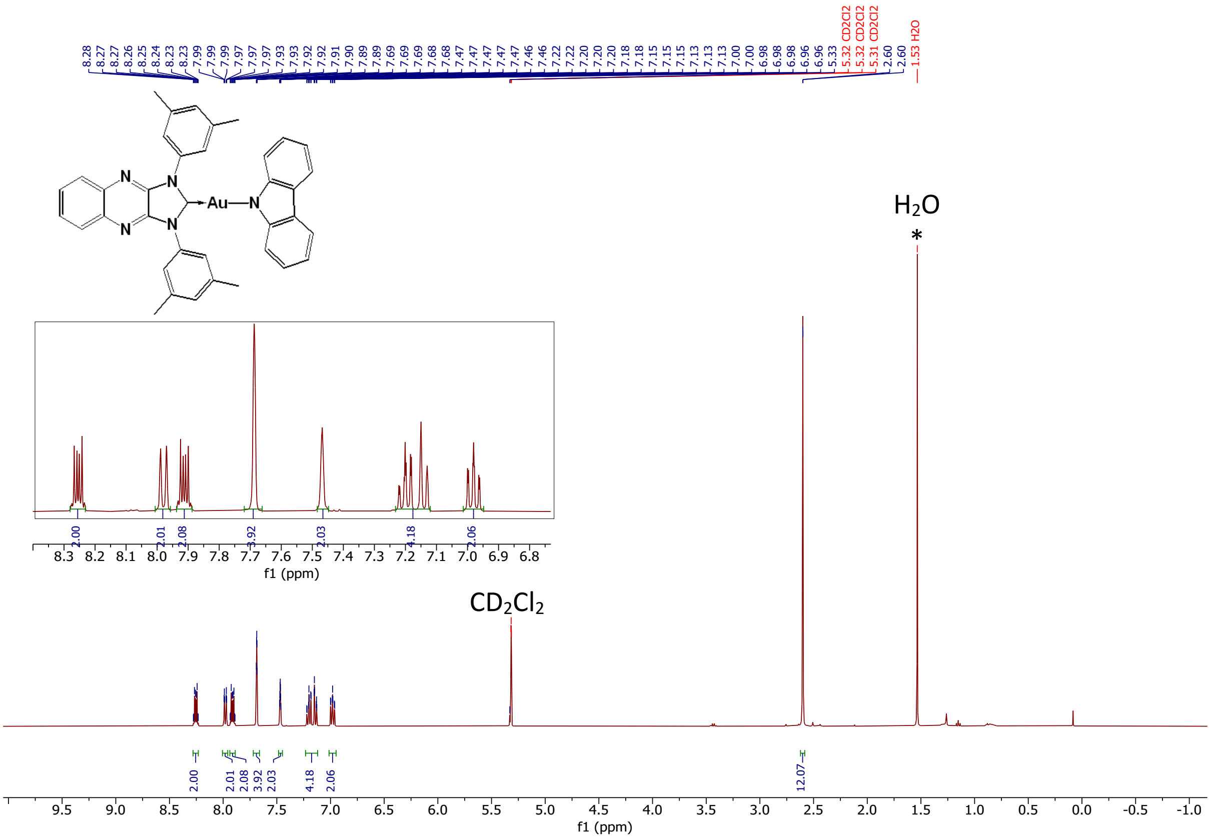

Figure S5. ^{1}H NMR (400 MHz, CD_2Cl_2)

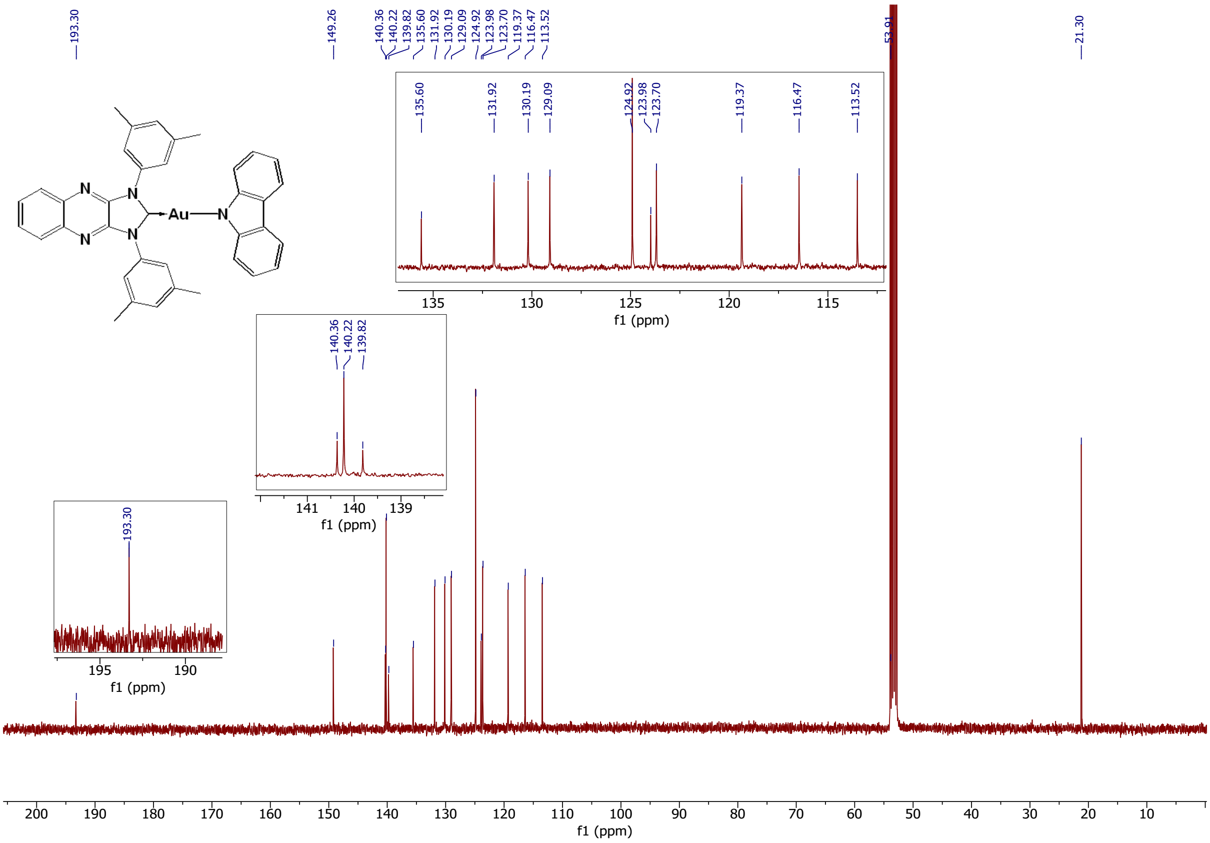

Figure S6. ^{13}C NMR (100 MHz, CD_2Cl_2)

Thermogravimetric Analysis.

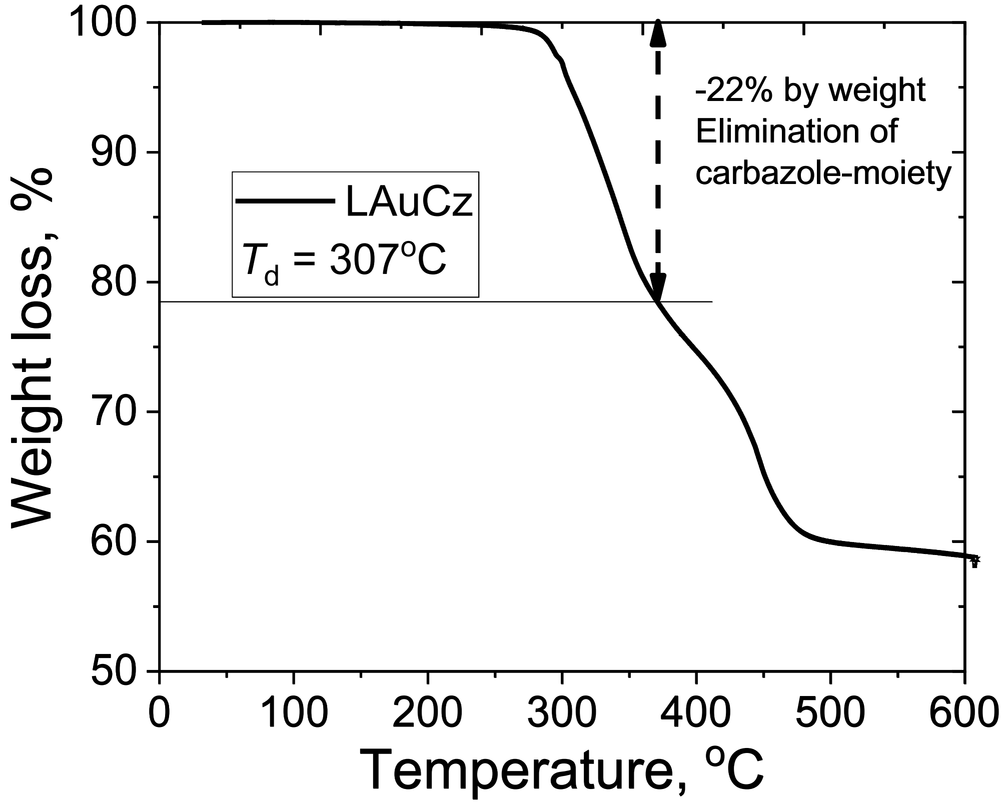

Figure S7. TGA curve for **LAuCz**, showing a stepwise decomposition. Decomposition temperature (T_d) indicates the temperature at 5% weight loss. The first decomposition step accounts for ca. 22% mass loss which can be attributed to the elimination of carbazole-moiety based on 21.6% molecular weight contribution.

X-Ray Crystallography.

Crystals suitable for X-ray diffraction study were obtained by slow layer diffusion of hexanes/petroleum ether into dichloromethane solution for **LAuCz** at room temperature. Crystals were mounted in oil on a MiTeGen loop and fixed on the diffractometer in a cold nitrogen stream. Data were collected using dual wavelength Rigaku FR-X rotating anode diffractometer using CuKα (λ = 1.54146 Å) radiation, equipped with an AFC-11 4-circle kappa goniometer, VariMAX^{TM} microfocus optics, a Hypix-6000HE detector and an Oxford Cryosystems 800 plus nitrogen flow gas system, at a temperature of 100K. Data were collected and reduced using CrysAlisPro v42.[1,2] Absorption correction was performed using empirical

methods (SCALE3 ABSPACK) based upon symmetry-equivalent reflections combined with measurements at different azimuthal angles. Molecules of **LAuCz** experiences a static disorder of the carbazolide ligand with equal occupancies over two positions. Structures were solved by direct method/intrinsic phasing and refined by the full-matrix least-squares against F^2. All non-hydrogen atoms were refined with anisotropic atomic displacement parameters. All hydrogen atoms were positioned geometrically and constrained to ride on their parent atoms with C-H = 0.95-1.00 Å, and U_{iso} = 1.2−1.5 U_{eq} (parent atom). All calculations were performed using the SHELXL software and Olex2 graphical user interface.[2,3]

Complex LAuCz, CCDC number 2431742, $C_{39}H_{34}AuCl_4N_5$ (M =911.48 g/mol). Monoclinic, space group $P2_1/c$ (no. 14), a = 7.4874(2) Å, b = 17.5679(7) Å, c = 27.6166(9) Å, β = 96.442(3)°, V = 3609.7(2) Å^3, Z = 4, T = 100.00(10) K, μ(Cu Kα) = 10.666 mm^{-1}, $Dcalc$ = 1.677 g/cm^3, 11870 reflections measured (5.974° ≤ 2Θ ≤ 158.132°), 11870 unique (R_{int} = 0.0638, R_{sigma} = 0.0760) which were used in all calculations. The final R_1 was 0.0742 (I > 2σ(I)) and wR_2 was 0.2224 (all data).

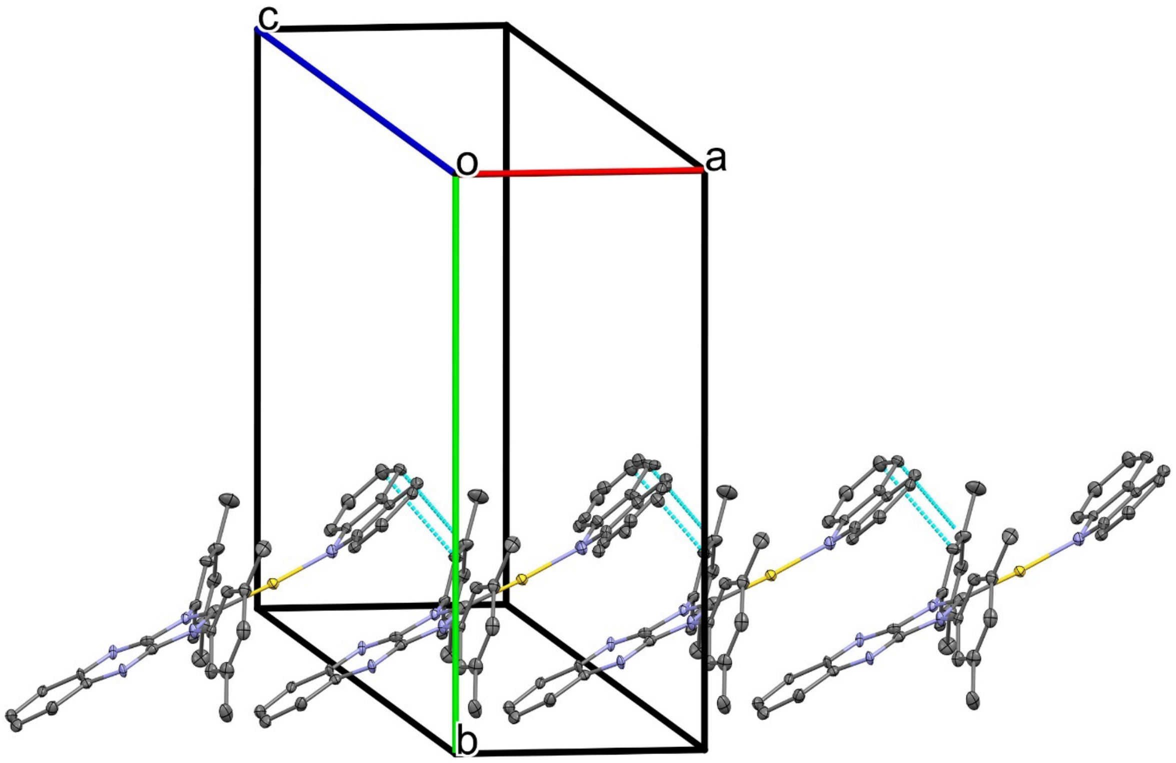

Figure S8. Molecular packing diagram for complex LAuCz along crystallographic axis *a*. Neighboring molecules of **LAuCz** are arranged in stacks via weak C–H(carbene)···π(amide) head to tail intermolecular interactions along crystallographic axis *a*.

Electrochemistry.

Cyclic voltammetry (CV) was performed using a three-electrode configuration consisting of a glassy carbon macrodisk working electrode (GCE) (diameter of 3 mm; BASi, Indiana, U.S.A.) combined with a Pt wire counter electrode (99.99%; GoodFellow, Cambridge, U.K.) and an Ag wire pseudoreference electrode (99.99%; GoodFellow, Cambridge, U.K.). The GCE was polished between experiments using alumina slurry (0.3 μm), rinsed in distilled water and subjected to brief sonication to remove any adhering alumina microparticles. The metal electrodes were then dried in an oven at 100 °C to remove residual traces of water, the GCE was left to air dry and residual traces of water were removed under vacuum. The Ag wire pseudoreference electrodes were calibrated to the ferrocene/ferrocenium couple in 1,4-difluorobenzene at the end of each run to allow for any drift in potential, following IUPAC recommendations.[4] All electrochemical measurements were performed at ambient temperatures under an inert N_2 atmosphere in tetrahydrofuran containing the compound under study (0.14 mM) and the supporting electrolyte [*n*-Bu_4N][PF_6] (0.13 mM). Data were recorded with Autolab NOVA software (v. 1.11).

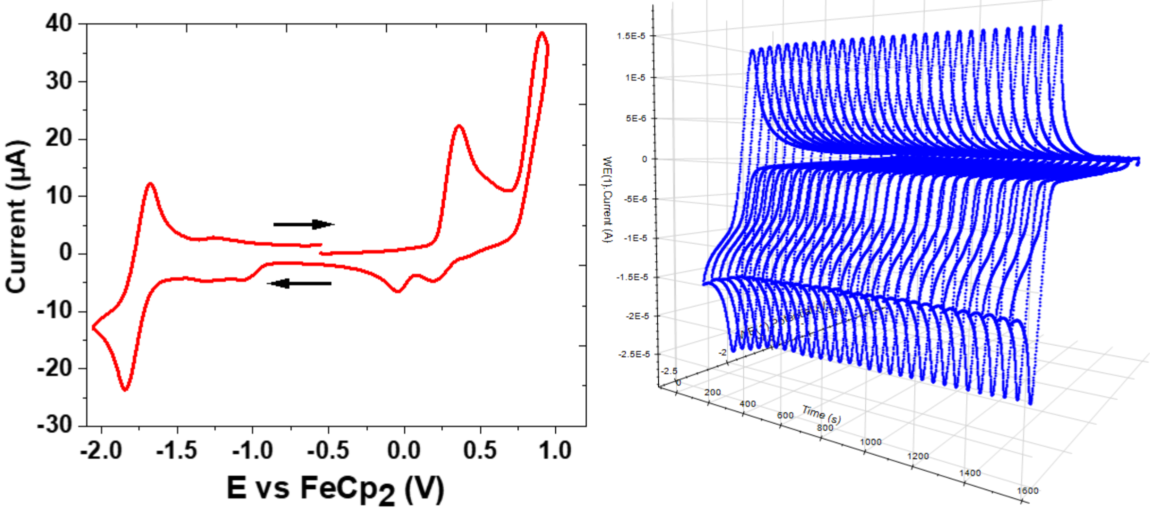

Figure S9. Full range cyclic voltammogram of complex **LAuCz**. Recorded using a glassy carbon electrode in THF solution (1.4 mM) with [n-Bu_4N]PF_6 as supporting electrolyte (0.13 M), scan rate 0.1 V s^{-1}. The near-unity ratio between cathodic and anodic currents i_{pc}/i_{pa} of 0.71 supports the quasi-reversible character of the reduction process (left) and remains unchanged after 30 reduction cycles indicating high electrochemical stability of the complex **LAuCz** (right).

Table S1. Selected distances [Å], angles [°],Formal electrode potentials (peak position E_p for irreversible and $E_{1/2}$ for quasi-reversible processes (*), V, *vs*. $FeCp_2$), onset potentials (E, V, *vs*. $FeCp_2$), peak-to-peak separation in parentheses for quasi-reversible processes (ΔE_p in mV), E_{HOMO}/E_{LUMO} (*e*V) and band gap values (ΔE, *e*V) for the redox changes exhibited by complexes **LAuCz** and **CMA1**.[a]

	Au–C, (Å)	Au–N, (Å)	C–Au–N, (°)	Torsion α, (°)	E_{ox} (V)	E_{HOMO} (eV)	E_{red} (V)	E_{LUMO} (eV)
**CMA1**	1.994(3)	2.027(2)	178.6(1)	17.1(1)	+0.25	–5.53	–2.80	–2.68
**LAuCz**	1.942(12)	2.022(15)	175.3(7)	16.5(3)	+0.36	–5.62	–1.76	–3.72

[a] torsion angle (α) $N–C_{carbene}\cdots N–C_{amide}$.

The torsion angle between the carbene and amide ligands is 16.5(1)°, which differs by less than 1° from that of the **CMA1** complex (Table S1) indicating a common two-coordinate linear geometry with co-planar arrangement of the ligands around gold(I) atom.

Experimental Methods for Spectroscopy

Sample Preparation

Samples for photophysical measurements were made from powders stored in a glovebox. Toluene solutions were made and kept in the glovebox until measured. Prior to measuring, solutions were transferred into 1mm path length quartz cuvettes which were sealed with a screw-top cap, parafilm and Teflon tape. Cuvettes were taken out of the glovebox immediately prior to experiments to minimize oxygen ingress.

Films were made from chlorobenzene solutions of polystyrene with **LAuCz** concentration at 20 mg/mL mixed in the appropriate ratio. Solution was drop-cast onto quartz substrates heated to 80°C or spin coated at 1500 rpm depending on the desired thickness. Films were made and stored in a glovebox. For transient absorption, films were encapsulated inside the glovebox using a coverslip, spacer tape and epoxy.

Time-Correlated Single Photon Counting

A 407nm PicoQuant LDH400 40MHz laser was used to photoexcite the samples. The repetition rate was set to 20-30kHz by a signal generator. A Princeton Instruments

SpectraPro2500i spectrograph with a CCD was used to collect emitted photons, and the time taken for the first photon to be emitted after excitation was recorded.

Photoluminescence Quantum Yield

Quantum yields have been measured in air for solid samples and under nitrogen for solutions. Photoluminescence quantum yields were recorded using an Hamamatsu Quantaurus-QY C11347-11. Quantum yields have been measured in air for solid samples and under nitrogen for solutions.

Steady-state Photoluminescence

Steady-state PL spectra were recorded using an Edinburg Instruments FLS1000 spectrofluorimeter. The light source was a monochromated 450 W Xenon arc lamp; excitation wavelength varied. The photostability experiments have been carried out with the excitation slit set to 1 for 375 nm wavelength. The light intensity was measured at ca. 46 $\mu W/cm^2$ with the ThorLabs S120VC standard photodiode power sensor. Samples were measured in air, vaccum or under flowing nitrogen at room temperature.

UV-Vis Absorption

UV-Vis spectra were measured using a Varian Cary 5000 UV-Vis-NIR spectrometer and Shimadzu UV-3600 Plus UV-VIS-NIR spectrophotometer. The spectrometer has a PMT detector for wavelength ranges from UV to visible, as well as InGaAs and PbS detectors for NIR. The light source used was a deuterium lamp for wavelengths less than 280nm and a tungsten halogen lamp for higher wavelengths.

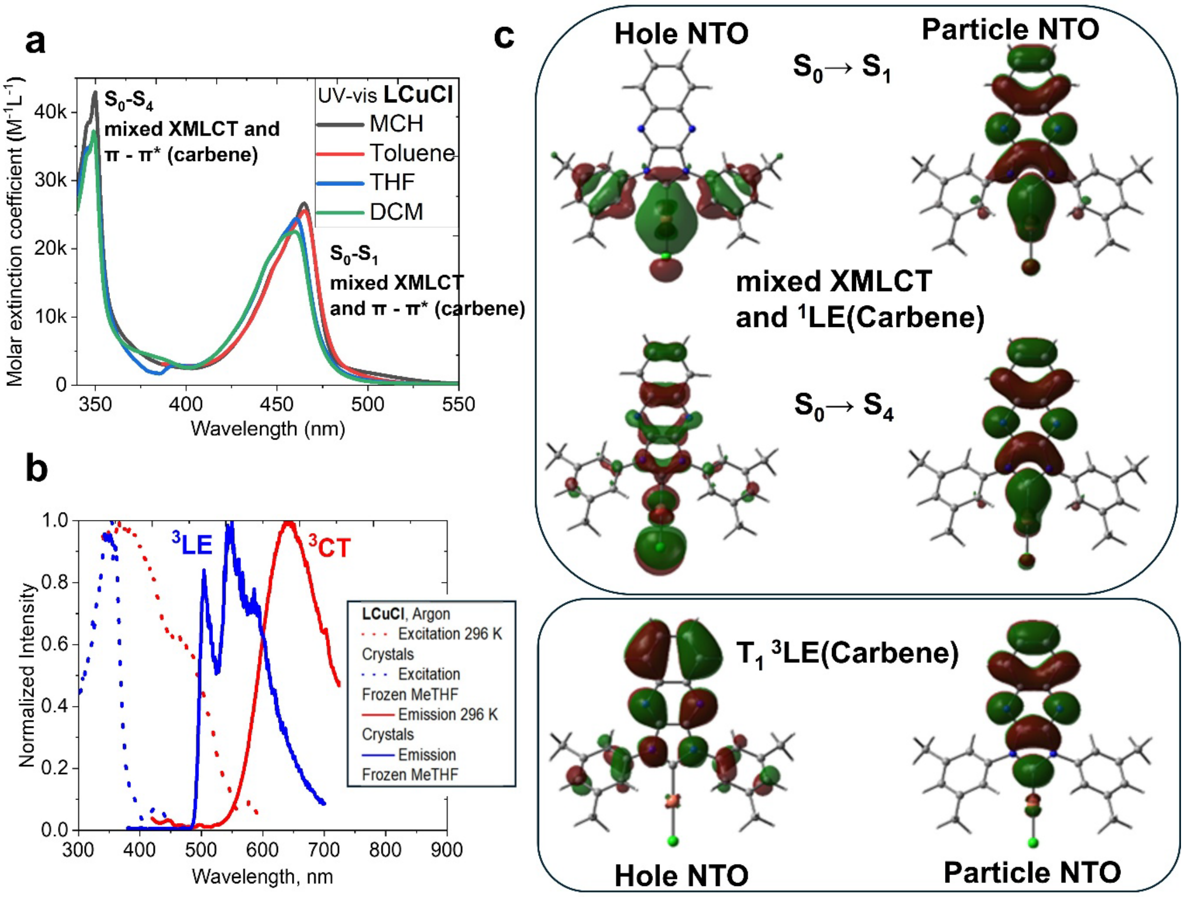

Figure S10. UV-vis, photoluminescence spectra and natural transition orbitals (NTOs) for copper complex LCuCl. **(a)** UV-vis absorption spectra in various solvents; **(b)** Photoluminescence spectra of **LCuCl** in frozen MeTHF (excitation at 365 nm); **(c)** Hole (left) and particle (right) NTO for **LCuCl**.

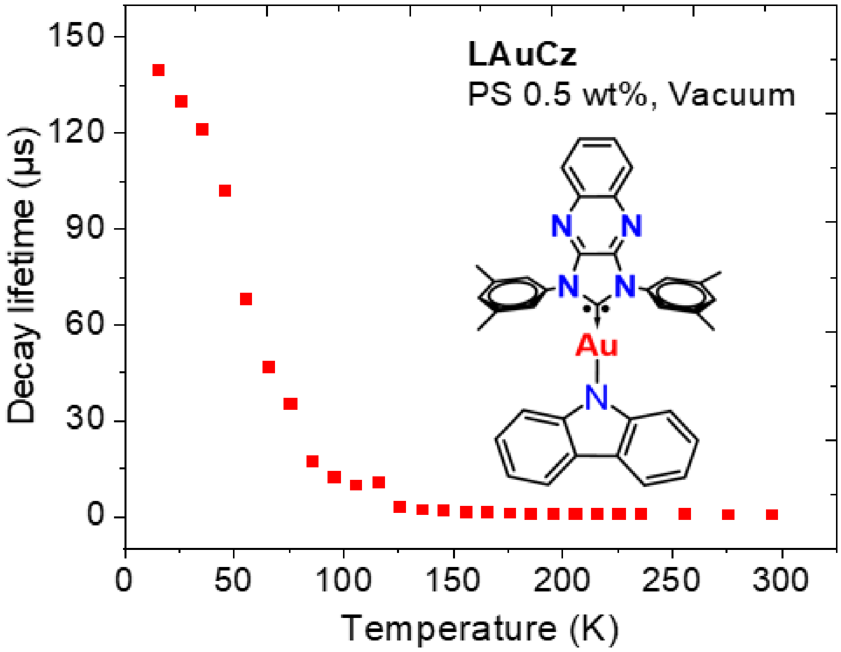

Figure S11. Varied-temperature excited state lifetime measurements for **LAuCz** in polystyrene matrix at 0.5% doping concentration.

Two-Photon Absorption

2PA measurements were performed at NanoMultiPhot (Institute of Molecular Science, Bordeaux university, Talence, France). The two-photon absorption were measured using the well-established method described by Xu and Webb.[6] An Ultra II (Coherent) femtoseconds pulsed laser (repetition rate: 80 MHz, pulsed width: 150 fs) was focalized on samples or reference solutions using an air Thorlabs objective (LMM15X-P01, 0.3 NA) under magnetic stirring. Excitation wavelength was tuned in the range 680-1050 nm and 1070-1200 nm (polystyrene matrix). Extended NIR-II wavelengths (*i.e.* 1070-1200 nm) were generated by pumping -with the Ultra II- an optical parametric oscillator (MIRA OPO Coherent) in linear configuration. Visible frequencies were removed using a 800 nm long-pass filter before exciting the sample. The 2PA measurements were conducted on Argon saturated solutions of **LAuCz** in toluene, THF (12 mM) and dispersed in polystyrene matrix (C= 1.4 mM) by two-photon excited fluorescence (TPEF) method, using fluorescein in aqueous NaOH (C= 0.22

mM, pH= 11.0) as two-photon reference.[5,6] For 2PA measurements on the range 1070 nm-1200 nm, Nile Red in DMSO (C= 97 μM) was used as two-photon reference.[7,8]
The 1 cm quartz cuvette -containing the solution or the PS matrix- was set in order to have the fluorescence generated on the close vicinity of the wall of the cuvette to limit reabsorption of fluorescence. The solution was stirred using magnetic bar to limit photobleaching during the measurements. The fluorescence is then collected in epifluorescence and filtered from back-scattered laser using a dichroic mirror (650 dcxru or 780 dcxruv, Chroma), filtered to remove residual laser scattering (FF01-650/SP-25, Semrock or E750sp-2p, Chroma) and finally send to a fast spectrophotometer (MayaPro, OceanInsight) by the mean of an optical fiber. The power of the laser is tuned using a polarizer at Brewster's angle (142013, Layertec) in tandem with a rotating halfwave plate. The power is measured in real time by sending a part of the laser beam on a silicon photodiode (S142CL with power meter interface PM101U, both from Thorlabs) using a beam splitter (UFBS5050, Thorlabs). Quadraticity of the TPEF signals is evaluated checking the quadratic dependence of fluorescence to the laser power. The full programs used for TPEF measurements and analysis are available on github.[7] The two-photon brightness $\sigma_2\Phi$ of samples were calculated using equation 1.

$$\sigma_2\Phi = \sigma_2\Phi_{ref} * \left(\frac{n}{n_{ref}}\right)^2 * \frac{C_{ref}}{C} * \left(\frac{\int Fluo.\ int.}{P^2} / \frac{\int Fluo.\ int._{ref}}{P_{ref}^2}\right) * \frac{\eta_{ref}}{\eta}$$

Equation 1. Calculation of two-photon brightness $\sigma_2\Phi$.

Where n is the refractive index, C the concentration, P the laser power, $\int Fluo.\ int.$ The fluorescence intensity and η the spectral correction of the sample and reference (*ca.* ref 7).

Sample preparation for 2-photon experiment. A quartz substrate was cleaned by sequential ultrasonication in chloroform, distilled water, acetone, isopropanol, and dried by isopropanol vapor. The substrate was then exposed to UV–ozone treatment for 15 min. In an argon-filled glovebox, 10 mg/ml chlorobenzene solution of polystyrene and LAuCz in the appropriate ratio was drop-cast onto the pre-cleaned quartz substrate. Then the substrate was annealed at 100 °C for 20 minutes to remove the residual solvent. The PS film was then placed in a 1 cm quartz cuvette and encapsulated before two-photon absorption measurements.

Table S2. Two-photon brightness ($\sigma_2\Phi$) and two-photon cross-section (σ_2) of **LAuCz** .

λ^{2PA} / nm	$\sigma_2\Phi$ / GM			σ_2 / GM		
	Toluene	THF	PS	Toluene	THF	PS
680	-	0.059	129	-	0.310	168
690	-	0.046	54.8	-	0.242	71.1
700	-	0.036	23.4	-	0.189	30.4
710	-	0.017	13.3	-	0.088	17.3
720	-	0.012	9.21	-	0.061	12
730	-	0.007	5.8	-	0.036	7.53
740	-	0.003	3.07	-	0.015	3.99
750	-	0.002	1.48	-	0.009	1.93
760	-	0.002	0.712	-	0.011	0.924
770	-	0.002	0.427	-	0.009	0.555
780	-	0.018	0.308	-	0.011	0.4
790	0.027	0.003	0.276	0.035	0.014	0.358
800	0.041	0.014	0.296	0.053	0.072	0.384
810	0.051	0.008	0.351	0.066	0.045	0.456
820	0.074	0.022	0.471	0.096	0.118	0.612
830	0.082	0.017	0.493	0.107	0.087	0.64
840	0.107	0.038	0.699	0.139	0.202	0.907
850	0.118	0.015	0.838	0.153	0.079	1.09
860	0.120	0.013	1.06	0.155	0.069	1.37
870	0.148	0.020	1.49	0.192	0.108	1.93
880	0.175	0.022	2.13	0.227	0.115	2.76
890	0.225	0.027	2.89	0.293	0.143	3.75
900	0.226	0.024	4.24	0.294	0.129	5.51
910	0.220	0.025	5.43	0.286	0.131	7.06
920	0.205	0.028	6.3	0.266	0.148	8.18
930	0.200	0.024	7.87	0.259	0.126	10.2
940	0.189	0.030	9.92	0.246	0.157	12.9
950	0.204	0.026	11.4	0.266	0.138	14.8
960	0.229	0.026	14.1	0.298	0.139	18.3

970	0.279	0.023	18.2	0.362	0.122	18.9
980	0.259	0.024	13.2	0.337	0.125	24.8
990	0.216	0.016	18.2	0.281	0.086	23.6
1000	0.146	0.011	13.2	0.189	0.058	17.1
1010	-	0.009	18.8	-	0.050	24.5
1020	-	0.010	21.1	-	0.055	27.4
1030	-	0.011	36.2	-	0.058	47
1040	-	0.012	46.6	-	0.064	60.5
1050	-	0.010	80.9	-	0.052	105
1070	-	-	73	-	-	94.8
1080	-	-	70	-	-	90.9
1090	-	-	61.4	-	-	79.7
1100	-	-	56.9	-	-	73.8
1110	-	-	49	-	-	63.6
1120	-	-	41.1	-	-	53.4
1130	-	-	25.6	-	-	33.2
1140	-	-	22.8	-	-	29.6
1150	-	-	22.8	-	-	29.6
1160	-	-	27.5	-	-	35.7
1170	-	-	23.9	-	-	31
1180	-	-	25.2	-	-	32.7
1190	-	-	12	-	-	15.6
1200	-	-	16.7	-	-	21.6

While the TADF emission exhibits a quadratic dependence on excitation power (confirming a second-order excitation process, *i.e.*, 2PA), the experimental absorption data in solution deviate from the theoretical calculations (Figure S15). Specifically, the 2PA response amplitude decreases by three orders of magnitude during the measurements. This discrepancy suggests the presence of an additional deactivation pathway occurring under 2PA measurements conditions. We hypothesize that, in solution, the high concentration of **LAuCz**, combined with the prolonged lifetime of the excited states, promotes the formation of non-fluorescent excimers or the occurrence of triplet-triplet annihilation processes. This assumption is support by the observation that restricting the diffusion of **LAuCz** (*i.e.* within a PS matrix) substantially restore the 2PEF response. Under these conditions, the allowed 2PA band (CT

$S_1 \rightarrow S_0$) appears distorted compared to one-photon excitation. This distortion may result from the depletion of the S_1 excited state, which is facilitated by long wavelength TADF emission, the use of a high-repetition-rate laser (80 MHz, 12.5 ns between pulses), the significantly longer excited-state lifetime of **LAuCz** (Table 2), and the viscosity of the host matrix which restricts orientation changes of the transition dipole moment.[8,9]

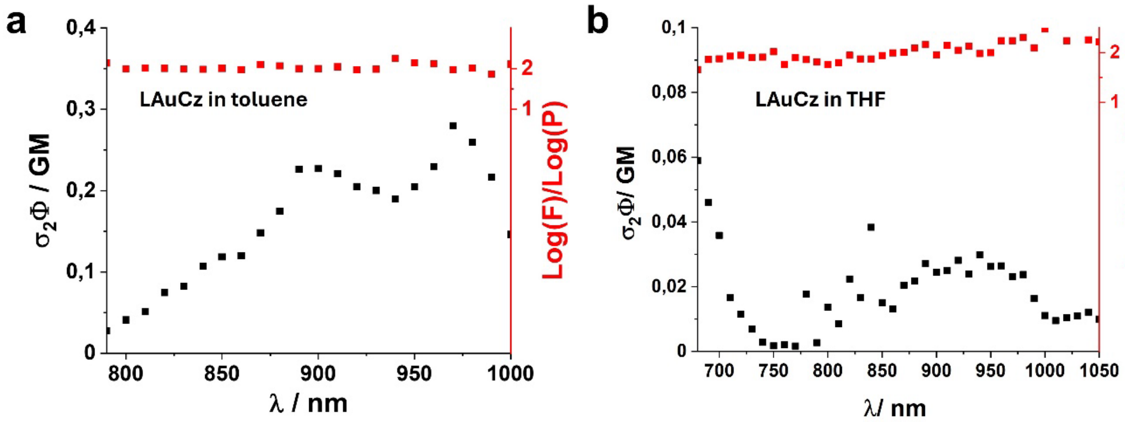

Figure S12. 1PA vs 2PA cross-sections (under Ar) spectra of **LAuCz** in solution (12 mM) in toluene (**a**) and THF (**b**).

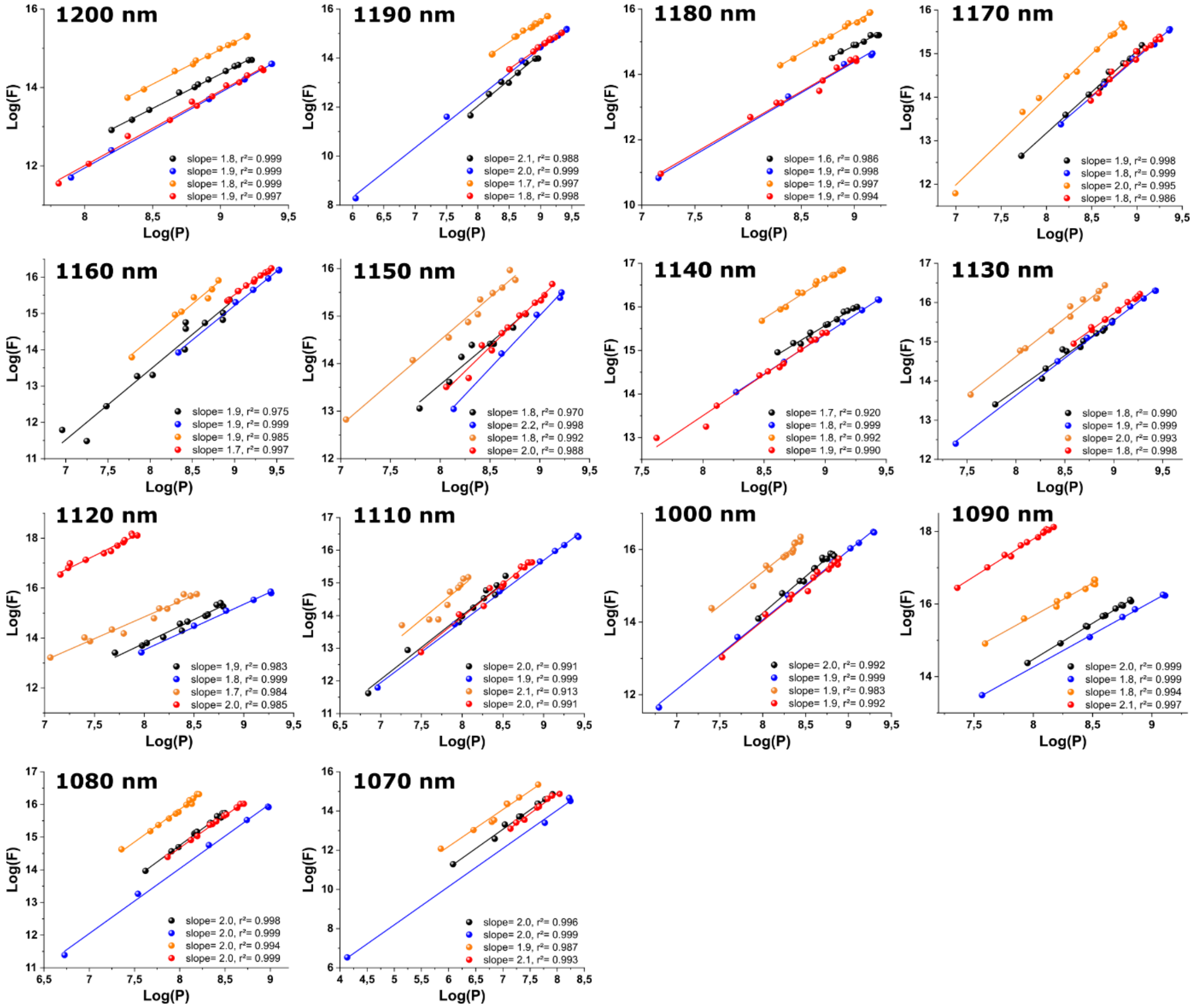

Figure S13. Two-photon process validation for 2P measures of **LAuCz** in polystyrene films with the OPO configuration ($\lambda^{ex.}$ = 1070-1200 nm). Measures were conducted on 4 different sites of the film (i.e. black, blue, orange and red). The quadratic dependence of the fluorescence (induced by 2P excitation) to the input mean power is measured at different excitation power for all excitation wavelengths. Only values that satisfy quadratic dependence are displayed.

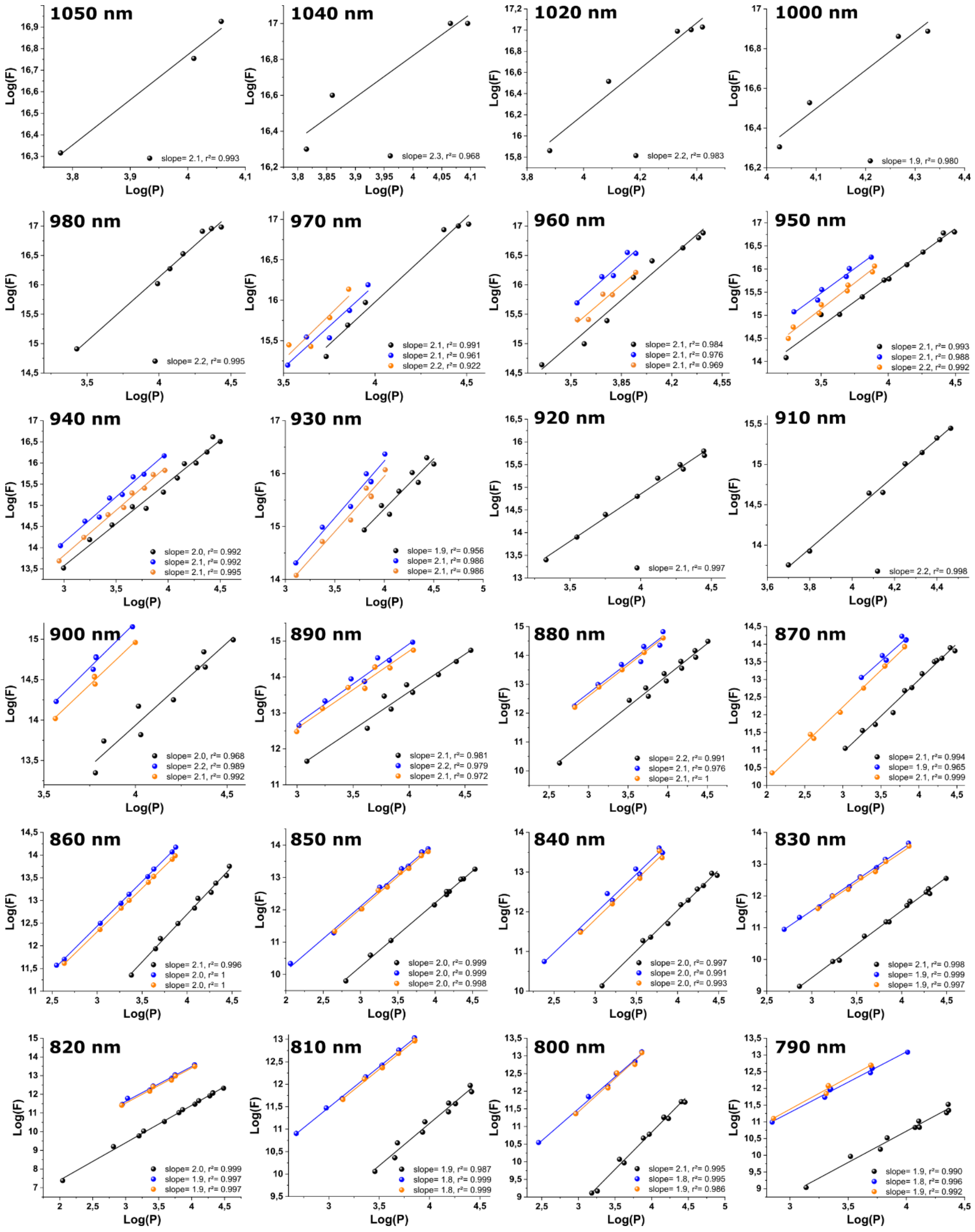

Figure S14. Two-photon process validation for 2P measures of LAuCz in polystyrene films with the NIR configuration ($\lambda^{ex.}$ = 1050-790 nm). Measures were conducted on 3 different sites of the film (i.e. black, blue and orange), except for $\lambda^{ex.}$ = 1050-980 nm. The quadratic dependence of the fluorescence (induced by 2P excitation) to the input mean power is measured

at different excitation power for all excitation wavelengths. Only values satisfying quadratic dependence (slope= 1.8-2.2) are displayed.

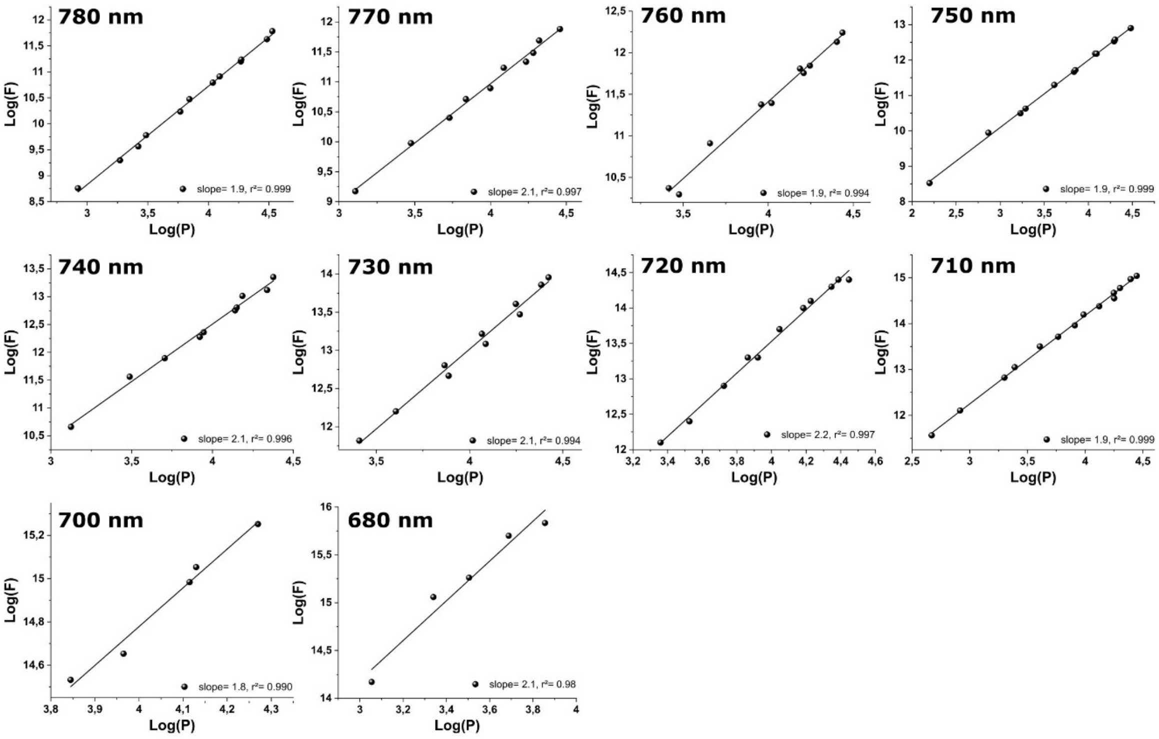

Figure S15. Two-photon process validation for 2P measures of **LAuCz** in polystyrene films with the NIR configuration ($\lambda^{ex.}$ = 780–680 nm). Measures were conducted on a single site of the film. The quadratic dependence of the fluorescence (induced by 2P excitation) to the input mean power is measured at different excitation power for all excitation wavelengths. Only values that satisfy quadratic dependence are displayed.

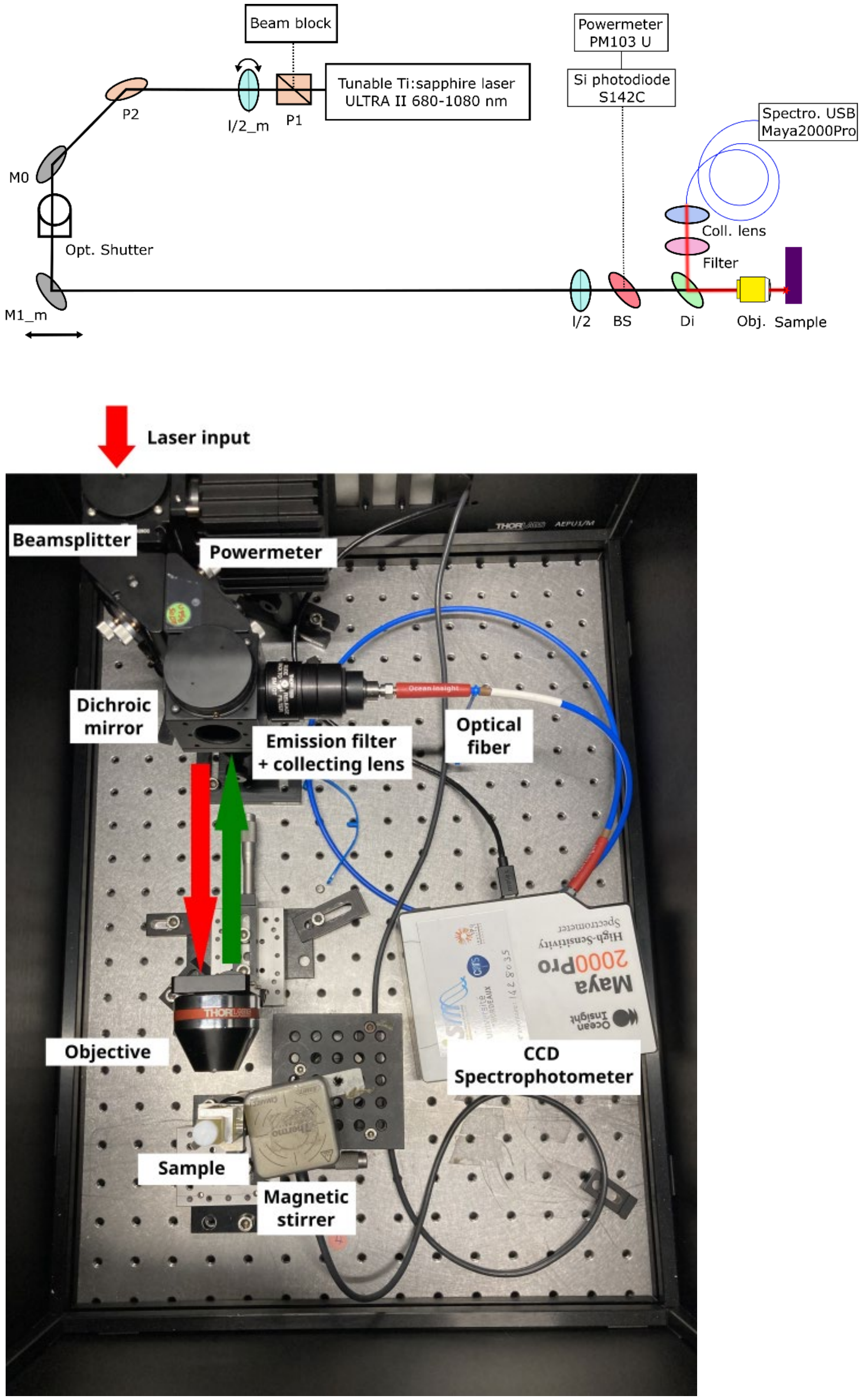

Figure S16. Top: NIR configuration (680-1050 nm) two-photon absorption measurement setup. P1: polarizing beamsplitting cube, l/2_m: motorized (Elliptec, #ELL14K) halfwave plate, P2: polarizer (LayerTec, #142013, 75°), M0: silver mirror (Thorlabs, #UM10-AG),

M1_m: motorized (Elliptec, #ELL20) silver mirror (Thorlabs, #UM10-AG), l/2: halfwave plate, BS: beamsplitter (Thorlabs, #UFBS 5050), Di: dichroic mirror (Chroma, #780 dcxruv or #650 dcxru), obj.: 15x, 0.3NA air objective (Thorlabs, #LMM15x-P01), Filter: emission filter (Chroma, #E750SP-2P), Coll. Lens: Air-Spaced doublet collimator (Thorlabs, #F810SMA-543), blue_line: optical fiber (OceanOptics, #P600-1-Vis-NIR). **Bottom:** photo of the 2P setup. Top view photo of the sample chamber of the two-photon excited fluorescence setup. Femtosecond laser beam is represented as a red arrow while fluorescence collected through the objective is represented as a green arrow. The focalisation of the laser beam in the sample (solution or film) is done just after the cuvette front face.

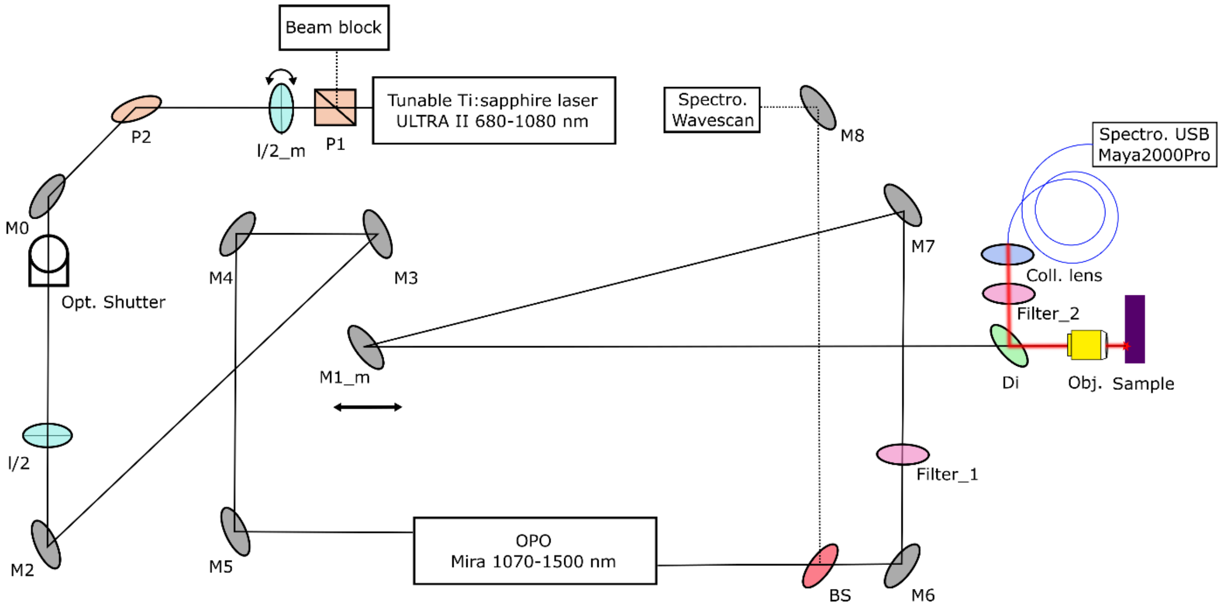

Figure S17. OPO configuration (1070-1200 nm) two-photon absorption measurement setup. P1: polarizing beamsplitting cube, l/2_m: motorized (Elliptec, #ELL14K) halfwave plate, P2: polarizer (LayerTec, #142013, 75°), M0-M8: silver mirrors (Thorlabs, #UM10-AG), M1_m: motorized (Elliptec, #ELL20) silver mirror (Thorlabs, #UM10-AG), l/2: halfwave plate, BS: beamsplitter (Thorlabs, #BSP10-B1), Filter_1: longpass filter (Schott, #SQ850 nm), Di: dichroic mirror (Chroma, #780 dcxruv or #650 dcxru), obj.: 15x, 0.3NA air objective (Thorlabs, #LMM15x-P01), Filter_2: emission filter (Chroma, #E750SP-2P), Coll. Lens: Air-Spaced doublet collimator (Thorlabs, #F810SMA-543), blue_line: optical fiber (OceanOptics, #P600-1-Vis-NIR).

Quantum chemical calculations

The ground state optimization for the complexes were studied by means of density functional theory (DFT) and the excited-state properties were computed at the time-dependent DFT (TD-DFT) level, using the Tamm-Dancoff approximation.[10,11] Calculations were carried by using the global hybrid MN15 functional of the Minnesota series by Truhlar and coworkers, which has especially good performance for noncovalent interactions and excitation energies.[12] The def2-TZVP basis set[13,14] was employed with relativistic effective core potential of 60 electrons for description of the core electrons of Au.[15] We have previously employed the selected methodology with success for closely related molecules.[16,17] Copper and gold metal contributions to HOMO and LUMO were calculated by the Mulliken population analysis and HOMO-LUMO overlap integrals were calculated using Multiwfn program.[18] All calculations were carried out with the Gaussian 16 suite package,[19] except the spin-orbit- coupling matrix elements which were calculated with Orca 5.0.4.[20]

Table S3. HOMO and LUMO for copper and gold complexes showing also contributions of the metal atomic orbitals.

	HOMO	LUMO
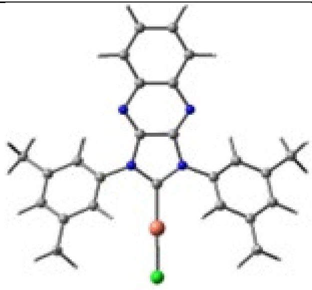<br>LCuCl<br>Overlap integral: 0.33	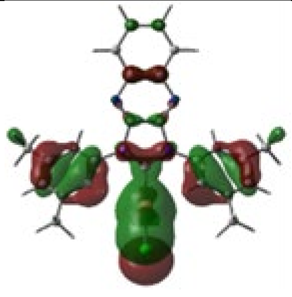<br>34.3%Cu	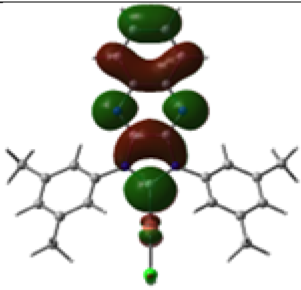<br>2.4%Cu
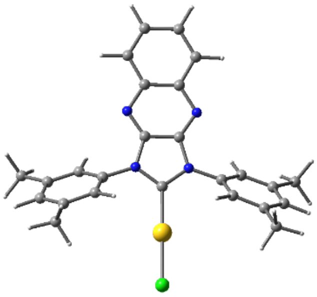<br>LAuCl<br>Overlap integral: 0.43	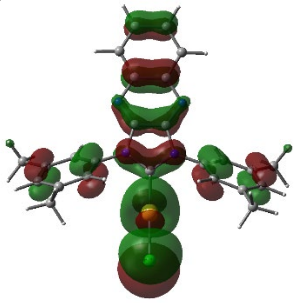<br>21.6%Au	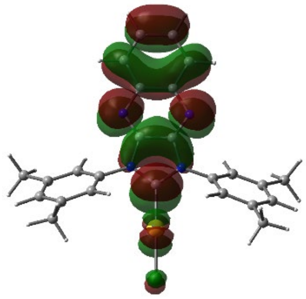<br>3.1%Au

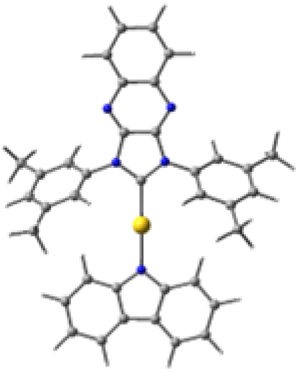 LAuCz Overlap integral: 0.26	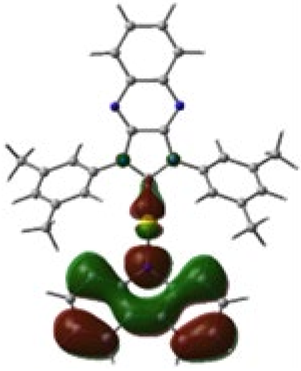 3.1%Au	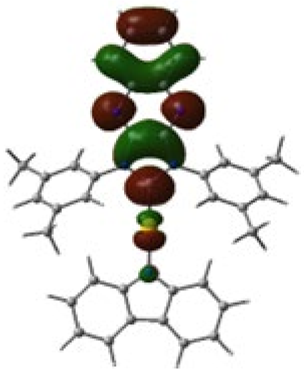 3.7%Au

Table S4. Calculated electric dipole moment (in Debye) of S_0 and S_1 states at the optimized S_0 geometry.

	S_0	S_1@S_0
LCuCl	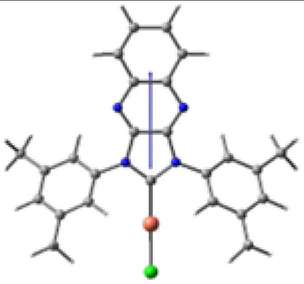 8.5D	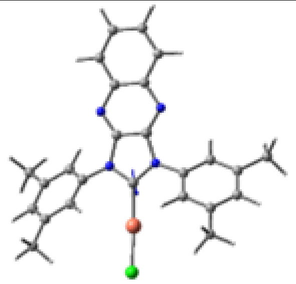 (-)2.4D
LAuCl	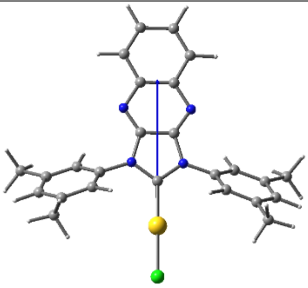 8.7D	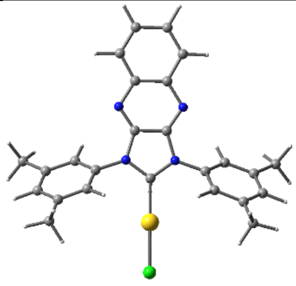 (-)1.4D
LAuCz	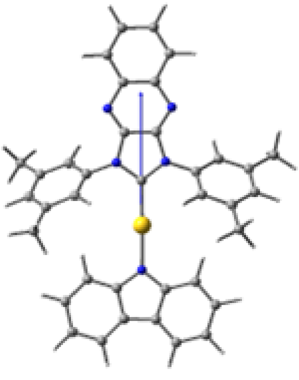 9.9D	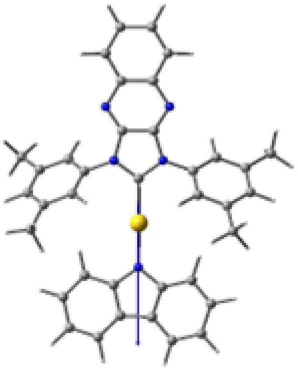 (-)13.0D

Table S5. Calculated vertical excitations, their orbital contributions, and their oscillator strengths (only for singlet excited states) .

	Excitation Energy	Orbital Contribution	Oscillator Strength
LCuCl	S_1 (mixed ^{1}CT and ^{1}LE(Carbene)): 3.31eV = 375nm	HOMO – LUMO (41%)<br>HOMO-2 – LUMO (39%)	0.008
	S_4 (mixed ^{1}CT and ^{1}LE(Carbene)): 3.80eV = 327nm	HOMO – LUMO (44%)<br>HOMO-2 – LUMO (43%)	0.291
	T_1 (^{3}LE(Carbene)): 2.97eV = 418nm	HOMO-3 – LUMO (48%)<br>HOMO-7 – LUMO (31%)	
	T_2 (mixed ^{3}CT and ^{3}LE(Carbene)): 3.04eV = 407nm	HOMO – LUMO (49%)<br>HOMO-2 – LUMO (17%)	
	T_3 (mixed ^{3}CT and ^{3}LE(Carbene)): 3.15eV = 394nm	HOMO-2 – LUMO (41%)<br>HOMO-6 – LUMO (19%)<br>HOMO-8 – LUMO (18%)	
LAuCl	S_1 (mixed ^{1}CT and ^{1}LE(Carbene)): 3.76eV = 330nm	HOMO-2 – LUMO (51%)<br>HOMO – LUMO (35%)	0.0557
	S_5 (mixed ^{1}CT and ^{1}LE(Carbene)): 3.97eV = 312nm	HOMO – LUMO (50%)<br>HOMO-2 – LUMO (33%)	0.4650
	T_1 (^{3}LE(Carbene)): 3.00eV = 413nm	HOMO-3 – LUMO (48%)<br>HOMO-6 – LUMO (32%)	
	T_2 (mixed ^{3}CT and ^{3}LE(Carbene)): 3.14eV = 394nm	HOMO – LUMO (54%)<br>HOMO-7 – LUMO (23%)	
	T_3 (^{3}LE(Carbene)): 3.54eV = 350nm	HOMO-9 – LUMO (66%)	

	S_1 (mixed ^{1}CT and ^{1}LE(Carbene)): 3.76eV = 330nm	HOMO-2 – LUMO (51%)<br>HOMO – LUMO (35%)	
LAuCz	S_1 (^{1}CT): 2.33eV = 532nm	HOMO – LUMO (98%)	0.260
	T_1 (^{3}CT): 2.13eV = 582nm	HOMO – LUMO (95%)	
	T_2 (^{3}CT): 2.88eV = 431nm	HOMO-1 – LUMO (97%)	
	T_3 (^{3}LE(Carbene)): 3.01eV = 412nm	HOMO-4 – LUMO (54%)<br>HOMO-8 – LUMO (23%)	

Table S6. Calculated hole-particle natural transition orbitals (NTOs) for the relevant excited states.

		Hole NTO	Particle NTO
LCuCl	S_1	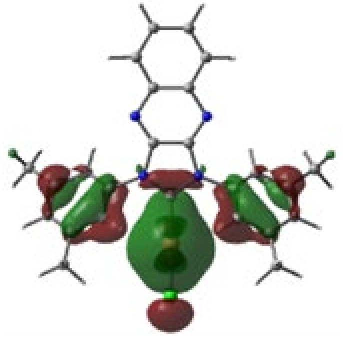	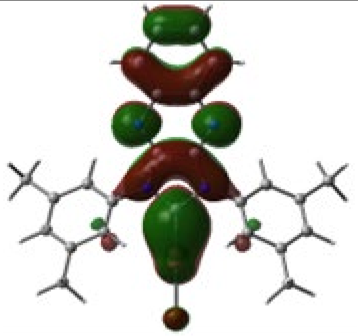
	S_4	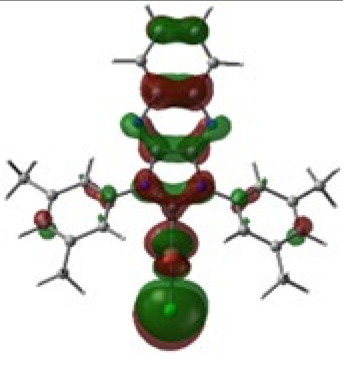	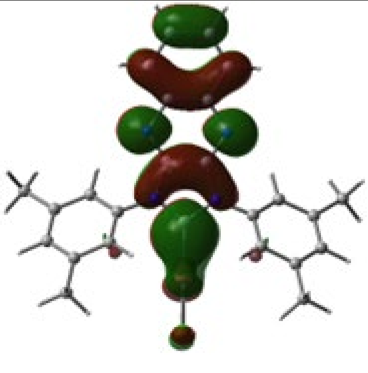
	T_1	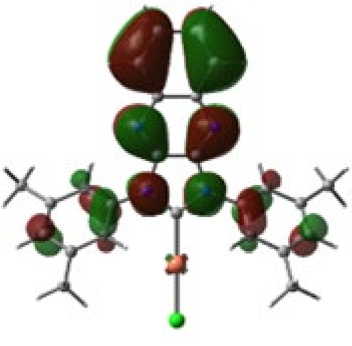	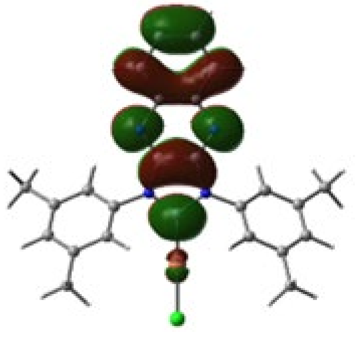

	T_2	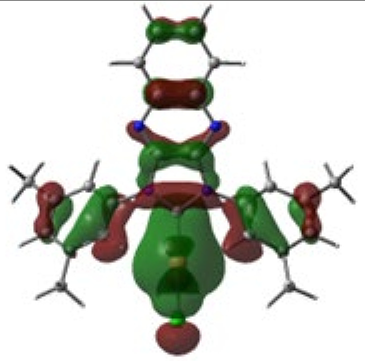	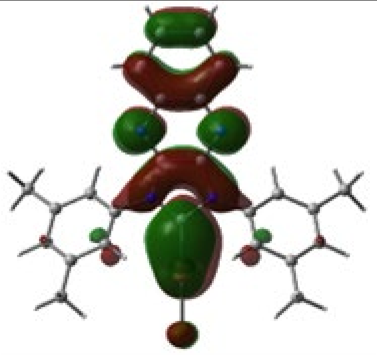
	T_3	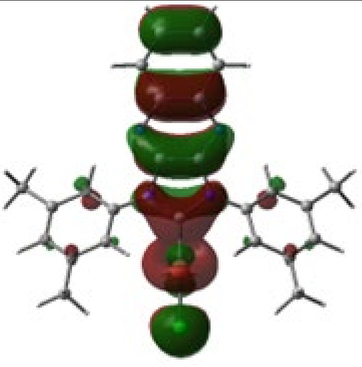	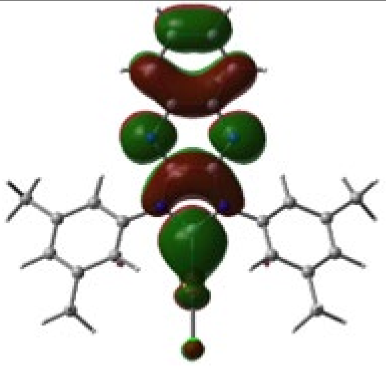
LAuCl	S_1	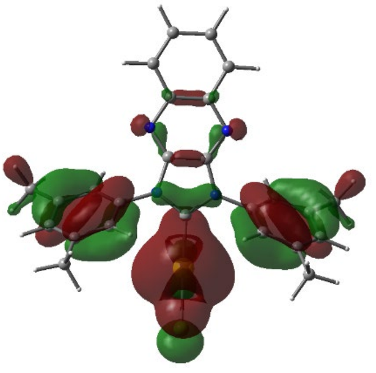	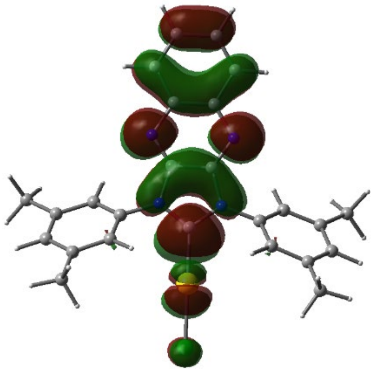
	S_5	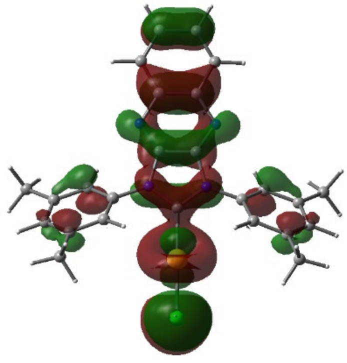	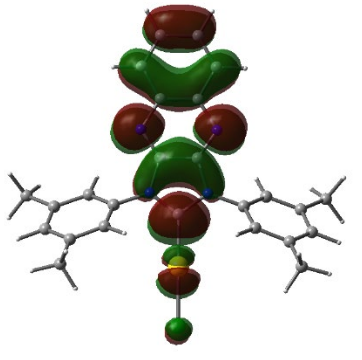

	T_1	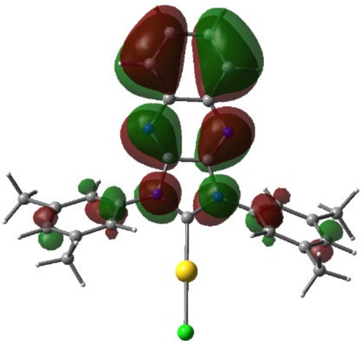	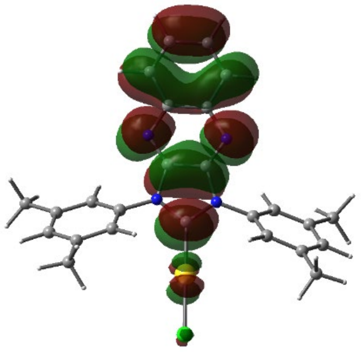
	T_2	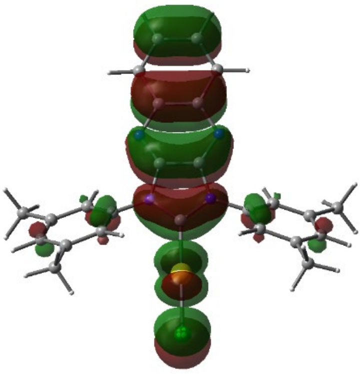	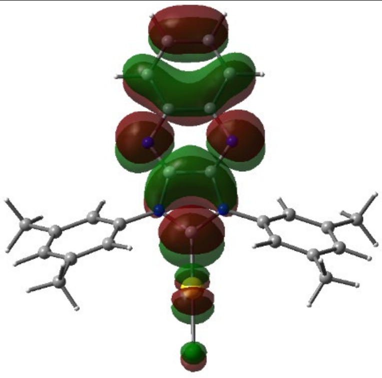
	T_3	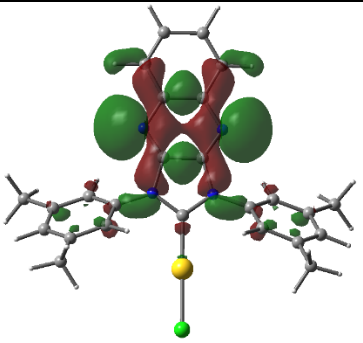	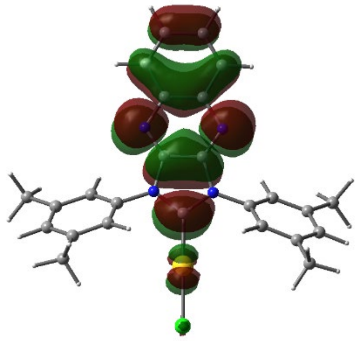
LAuCz	S_1	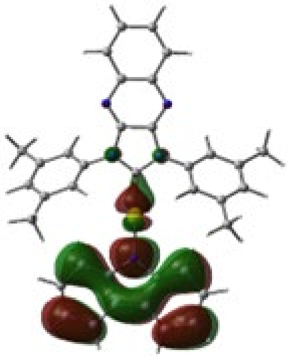	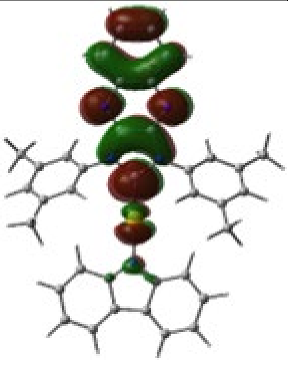

	T_1	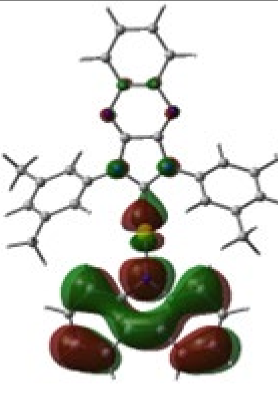	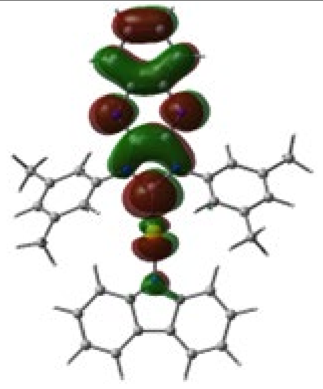
	T_2	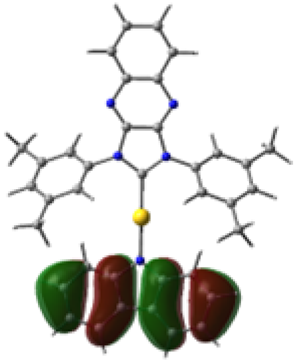	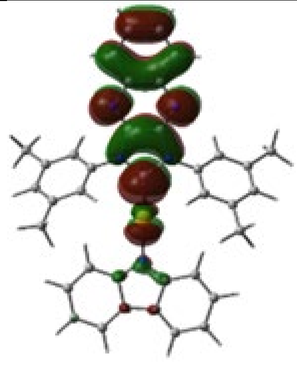
	T_3	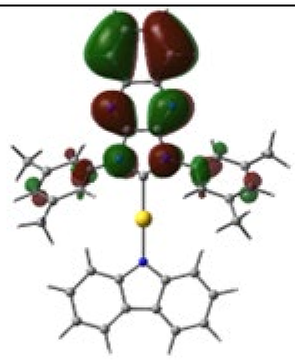	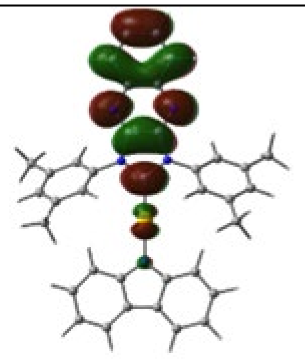

Table S7. Calculated spin-orbit- coupling matrix elements (SOCME, cm^{-1}) for complex **LAuCz**. (MN15/def2-TZVP).[18]

	State		Component			SOCME
	T	S	Z	X	Y	cm^{-1}
LAuCz	1	0	-0.75	0.92	1.81	2.16
	1	1	-0.03	0.01	-6.07	6.07
	1	2	-24.58	10.79	-1.53	26.88
	1	3	-4.59	8.79	25.23	27.10
	2	0	-30.94	-1.65	-4.54	31.31
	2	1	-20.37	11.76	-0.81	23.53
	2	2	-0.1	0.6	-1.2	1.34
	2	3	9.25	-5.55	0.31	10.79
	3	0	-17.46	-22.89	2.27	28.87

	3	1	-15.09	3.26	3.82	15.90
	3	2	-0.07	-0.79	1.42	1.62
	3	3	0.76	1.07	-0.85	1.56

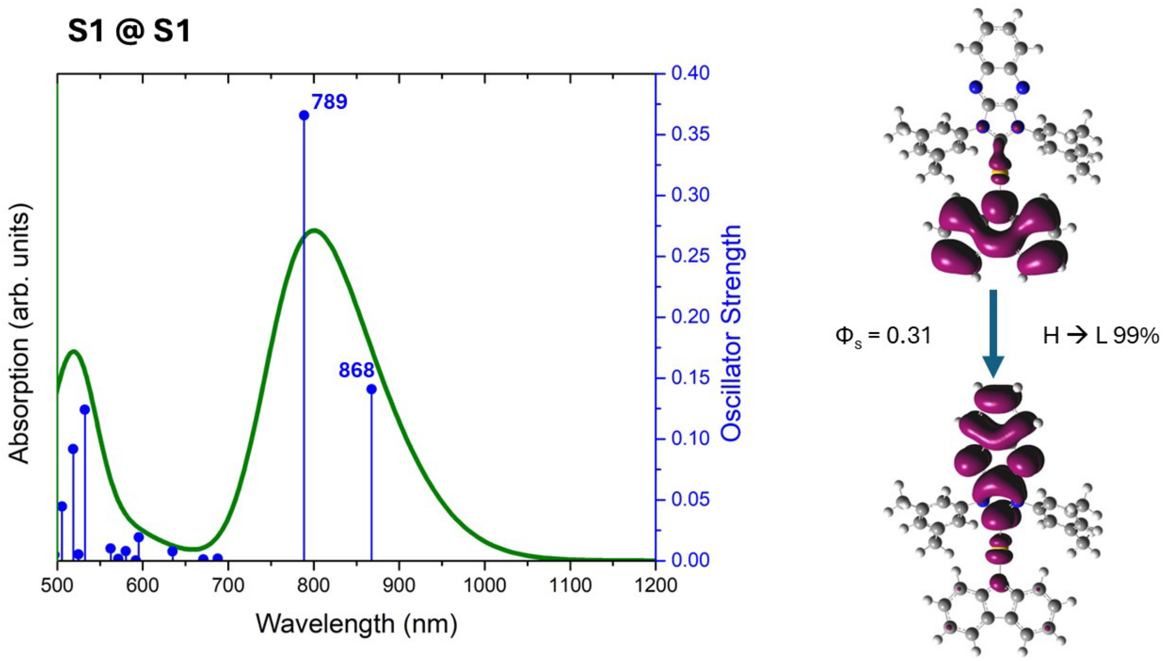

Figure S18: LAuCz excited-state absorption of S1 at its optimized excited-state geometry. The hole-electron densities are reported as well, along with the Φ_s metric and the orbital contribution for such transition.

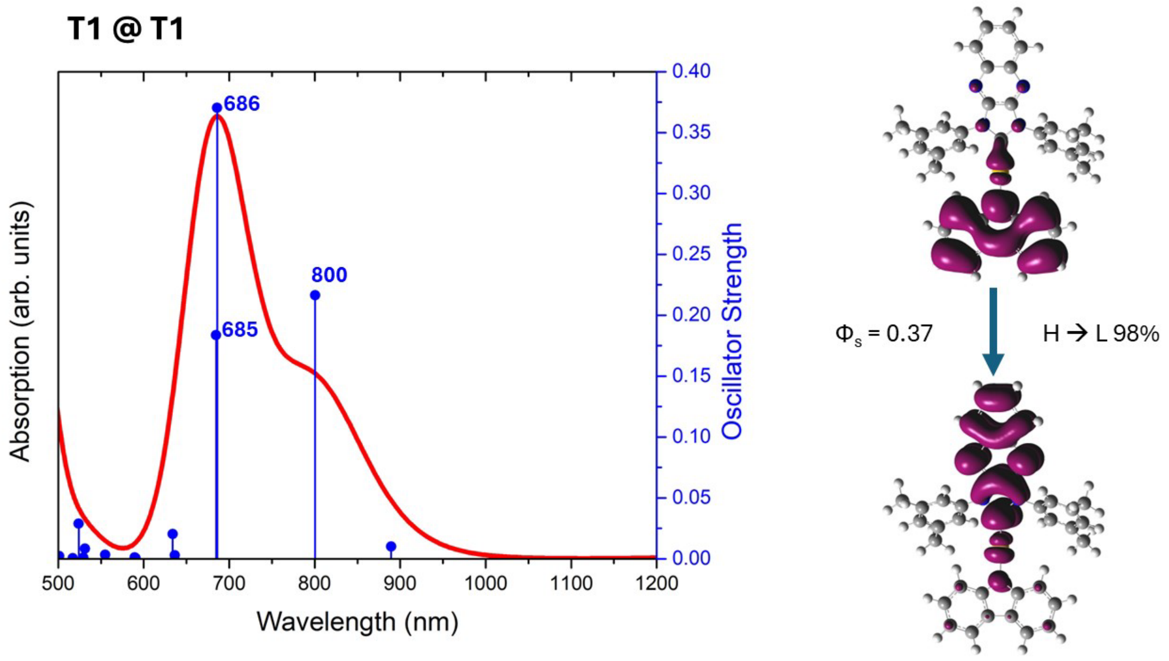

Figure S19: LAuCz excited-state absorption of T1 at its optimized excited-state geometry. The hole-electron densities are reported as well, along with the Φ_s metric and the orbital contribution for such transition.

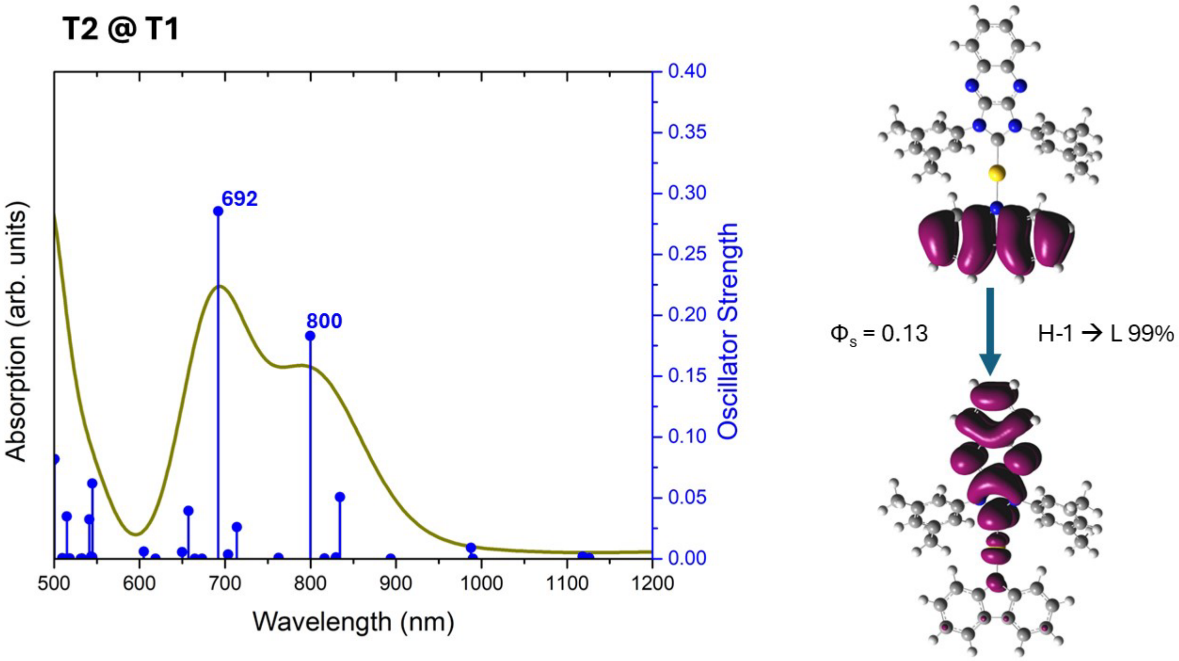

Figure S20: LAuCz excited-state absorption of T2 at the T1 optimized excited-state geometry. The hole-electron densities are reported as well, along with the Φ_s metric and the orbital contribution for such transition.

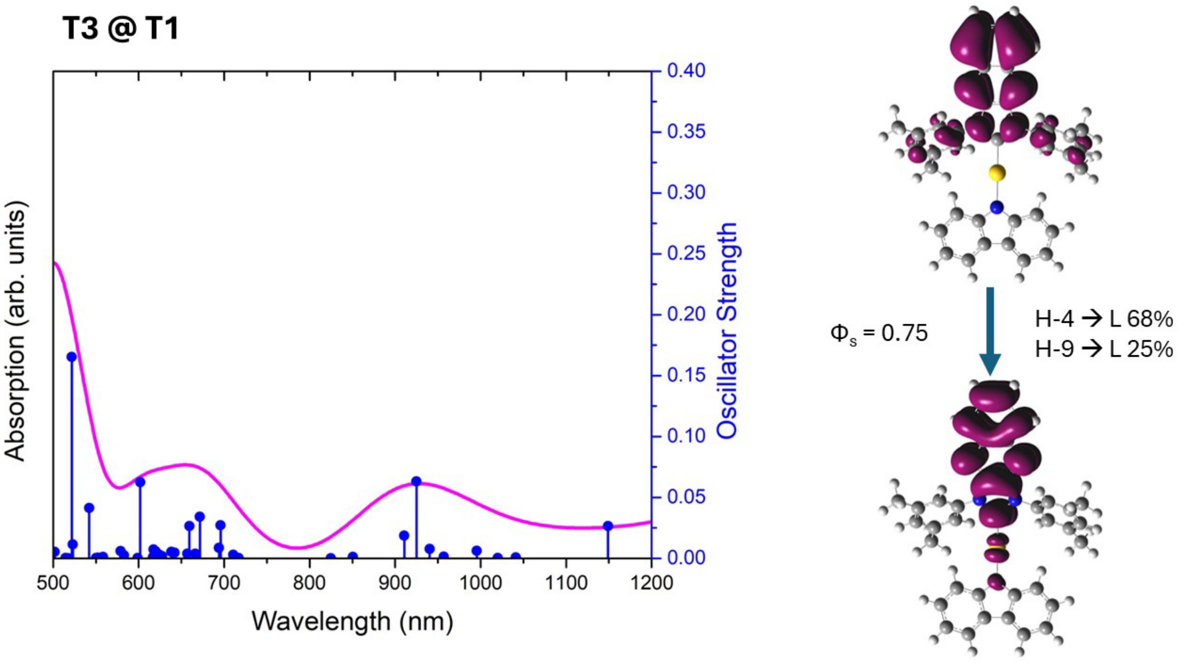

Figure S21: LAuCz excited-state absorption of T2 at the T1 optimized excited-state geometry. The hole-electron densities are reported as well, along with the Φ_s metric and the orbital contribution for such transition.

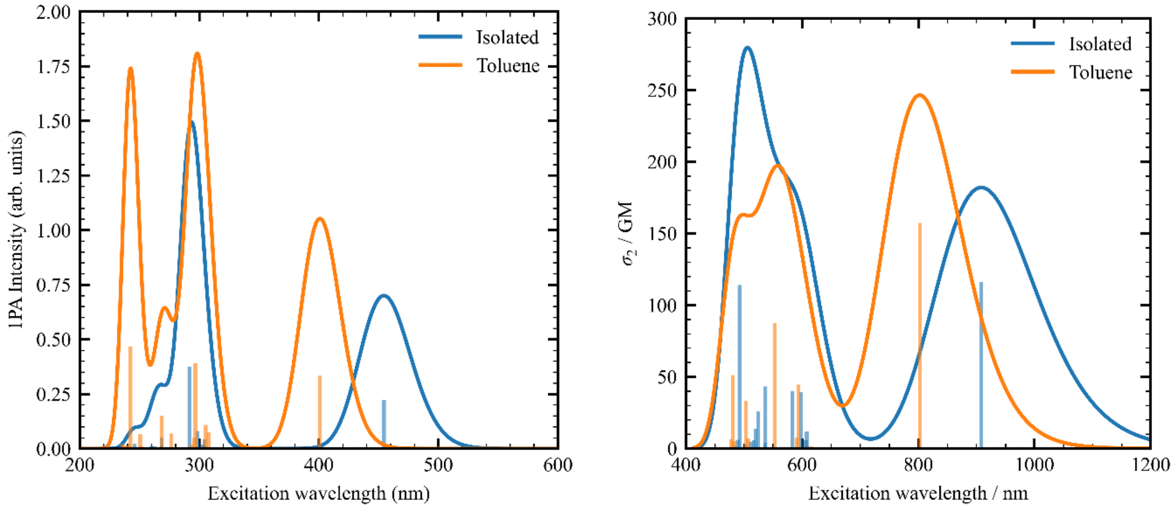

Figure S22: 1PA (left) and 2PA (right) absorption spectra of LAuCz considering the 20 lowest-lying transitions and 0.3 eV FWHM value.

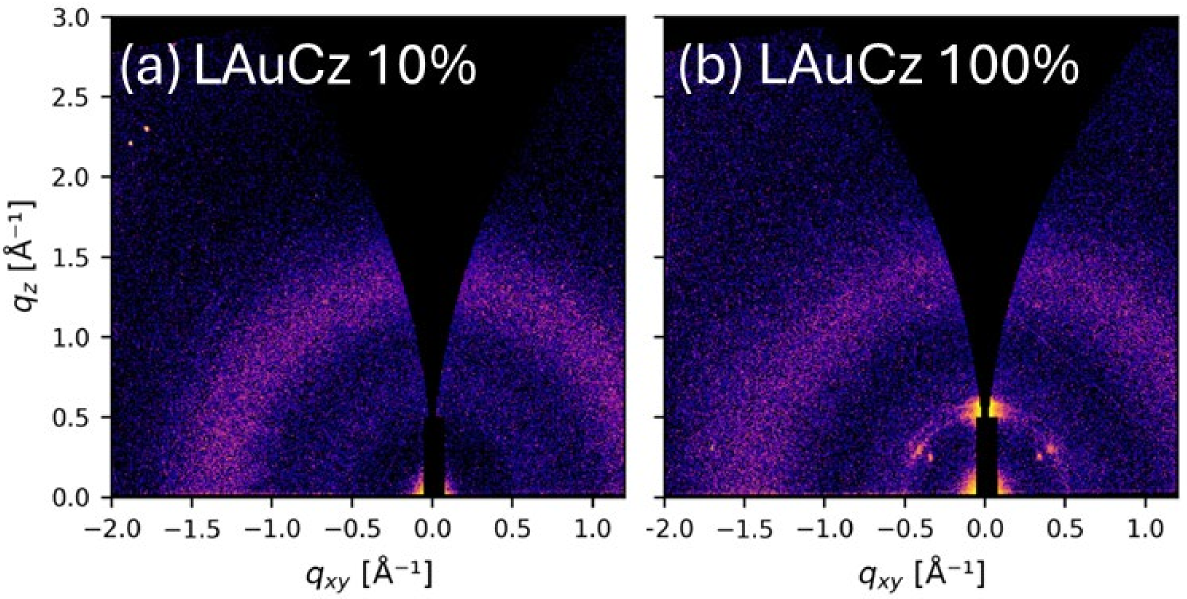

Figure S23. 2D GIWAXS patterns of: (a) **LAuCz** complex in 10 wt.% PS thin film; (b) **LAuCz** neat thin film. The intensity of X-ray scattering is presented on a common logarithmic colour scale.

Experimental methods of grazing incidence wide angle X-ray scattering measurements

GIWAXS measurements were performed using a Xeuss 3.0 laboratory beamling (Xenocs) equipped with a liquid gallium MetalJet source (Excillum), producing X-rays with an energy of 9.243keV (λ=1.34Å). A collimated X-ray beam was directed at sample surfaces inclined at a grazing angle of 0.15° and scattered X-rays were detected by a Pilatus3R 1M 2D X-ray detector (Dectris) positioned ~100 mm from the sample centre. The sample to detector distance was calibrated using a silver behenate standard in transmission geometry. GIWAXS data were corrected, reshaped and reduced using code based on pyfai and pygix python libraries. 1D intensity profiles were generated by azimuthally integrating the 2D patterns as a function of q = 4πsinθ/λ where 2θ is the angle between the incident and scattered X-ray of wavelength λ. Integrations were performed across the full q range.

Table S8. 1PA and 2PA absorption as obtained for LAuCz isolated and in toluene.

	Isolated			Toluene		
State	Transition Energy [eV]	1PA Intensity (arb. units)	σ [GM]	Transition Energy [eV]	1PA Intensity (arb. units)	σ [GM]
1	2.73	1.00	183	3.09	1.00	247
2	3.46	0.00	0	3.92	0.00	0
3	4.07	0.19	2	4.03	0.22	2
4	4.08	0.06	19	4.06	0.32	0
5	4.09	0.09	9	4.18	1.17	70
6	4.15	0.36	62	4.20	0.15	12
7	4.18	0.00	0	4.23	0.06	1
8	4.25	1.68	63	4.49	0.21	138
9	4.60	0.00	1	4.61	0.45	0
10	4.63	0.23	7	4.66	0.01	0

Table S9. S_0-S_1 2PA absorption cross-section as obtained using few-state models (equation 4) for LAuCz isolated and in toluene.

	Isolated	Toluene
States	σ [GM]	σ [GM]
S_0, S_1	260	426
S_0, S_1, S_2	260	426
S_0, S_1, S_3	260	428
S_0, S_1, S_4	253	422
S_0, S_1, S_5	265	333
S_{0-5}	258	330

Table S10. Permanent (i = j) and transition dipole moments as obtained for LAuCz isolated and in toluene.

Isolated					Toluene				
i	j	μ_x	μ_y	μ_z	i	j	μ_x	μ_y	μ_z
0	0	4.015	0.006	0.203	0	0	4.521	0.004	0.212
0	1	-1.830	-0.003	-0.093	0	1	-2.106	-0.006	-0.114
0	2	0.000	-0.008	0.002	0	2	0.001	0.051	-0.012
0	3	0.006	0.647	-0.151	0	3	-0.009	-0.866	0.199
0	4	-0.352	-0.032	-0.114	0	4	1.045	0.026	0.157
0	5	-0.437	0.045	0.153	0	5	-1.957	0.007	-0.054
1	1	-5.490	-0.020	-0.325	1	1	-6.035	-0.046	-0.453
1	2	-0.002	-0.193	0.044	1	2	0.004	0.217	-0.049
1	3	-0.025	-0.152	0.035	1	3	-0.004	-0.206	0.047
1	4	1.311	-0.013	0.007	1	4	-0.185	0.007	0.023
1	5	-0.684	0.014	0.029	1	5	2.263	0.012	0.147
2	2	-7.195	-0.035	-0.464	2	2	-8.124	-0.069	-0.640
2	3	-0.140	0.000	-0.010	2	3	1.056	0.007	0.073
2	4	-0.002	-0.028	0.006	2	4	0.005	-0.023	0.006
2	5	0.002	0.066	-0.015	2	5	-0.004	0.011	-0.003
3	3	4.093	0.020	0.201	3	3	5.025	0.007	0.222
3	4	-0.026	0.113	-0.027	3	4	0.001	0.061	-0.014
3	5	0.048	-0.559	0.130	3	5	-0.002	0.411	-0.094
4	4	2.871	0.000	0.136	4	4	5.244	0.015	0.299
4	5	-1.457	-0.011	-0.072	4	5	0.027	0.002	0.012
5	5	-0.068	0.020	0.122	5	5	4.324	0.005	0.205

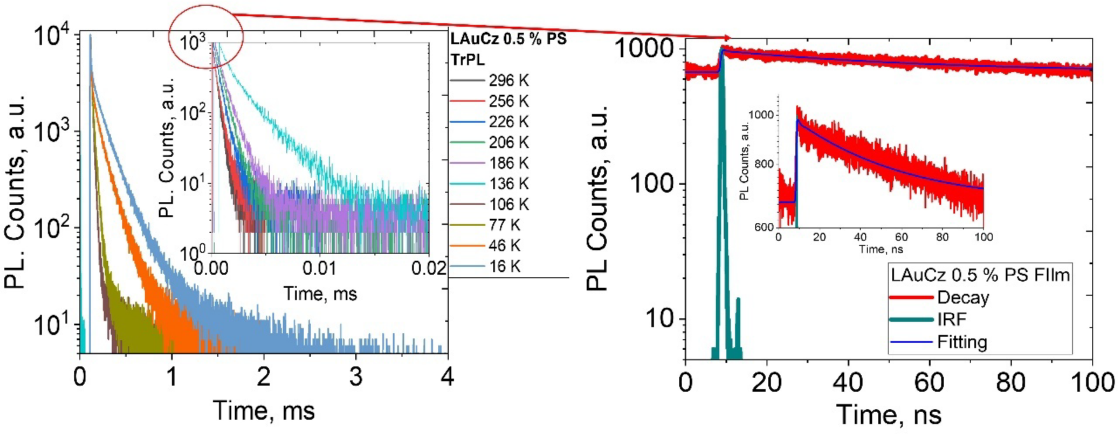

Figure S24. Varied temperature TCSPC traces in microsecond range for delayed emission (left) and nanosecond range prompt emission component at 296 K (right).

Table S11. Kinetic analysis for a TADF was performed according to three-state model for complex **LAuCz** in PS film at 0.5 wt%.[21]

	λ_{em} (nm)	τ_p (ns)	τ_d (μs)	Φ (%)	k_r^S (10^6 s^{-1})	k_{nr}^S (10^6 s^{-1})	k_{nr}^T (10^6 s^{-1})	k_{ISC} (10^8 s^{-1})	k_{RISC} (10^9 s^{-1})	S_1/T_1 (eV)^e
LAuCz	613	0.6	0.354	77	2.18	0.65	0.65	8.34	1.67	2.32/2.25

$$\phi_{PF} = \frac{k_d(A_p + A_d)}{A_p k_d + A_d K_p}\phi_{PLQY}$$

$$\phi_{DF} = \frac{A_d(k_p - k_d)}{A_p k_d + A_d k_p}\phi_{PLQY}$$

$$k_r^S = k_p \phi_{PF}$$

$$k_{nr}^{S^{MAX}} = k_p \frac{\phi_{PF}}{\phi_{PLQY}}(1 - \phi_{PLQY})$$

$$k_{ISC}^{Ave.} = \frac{[k_p(1-\phi_{PF}) - k_d\phi_{DF}]\phi_{PLQY} + k_p\phi_{PF}\phi_{PF} \pm [k_p\phi_{PF}^2(1-\phi_{PLQY}) + k_d\phi_{DF}\phi_{PLQY}]}{2\phi_{PF}\phi_{PLQY}}$$

$$k_{nr}^{T^{MAX}} = k_d(1 - \frac{\phi_{DF}}{1-\phi_{PF}})$$

$$k_{RISC}^{Ave.} = \frac{k_d}{2} \cdot \frac{\phi_{PLQY}(1-\phi_{PF}) + \phi_{DF} \pm \phi_{PF}(1-\phi_{PLQY})}{\phi_{PF}(1-\phi_{PF})}$$

where

A_p and A_d are the amplitudes of the prompt and delayed components of the emission lifetime, respectively.

ϕ_{PF} and ϕ_{DF} are the prompt and delayed fluorescence quantum yields, respectively.

k_p and k_d are rates of the prompt and delayed components.

k_r^S is radiative rate from the singlet state

k_{nr}^{SMAX} and k_{nr}^{TMAX} are the maximum non-radiative decay rates from the singlet and triplet states, respectively.

$k_{ISC}^{Ave.}$ and $k_{RISC}^{Ave.}$ are the average intersystem and reverse intersystem crossing rates, respectively.

a)

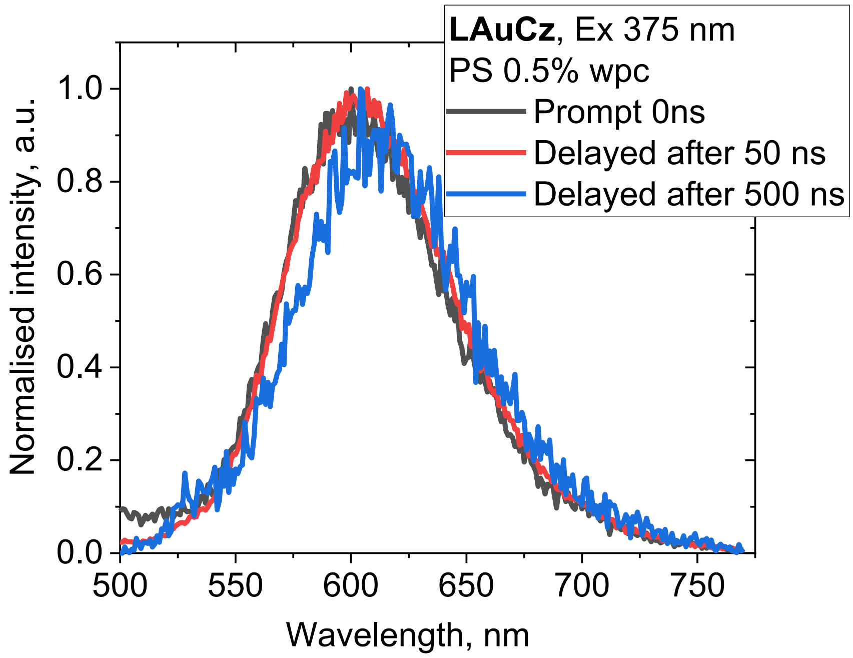

b)

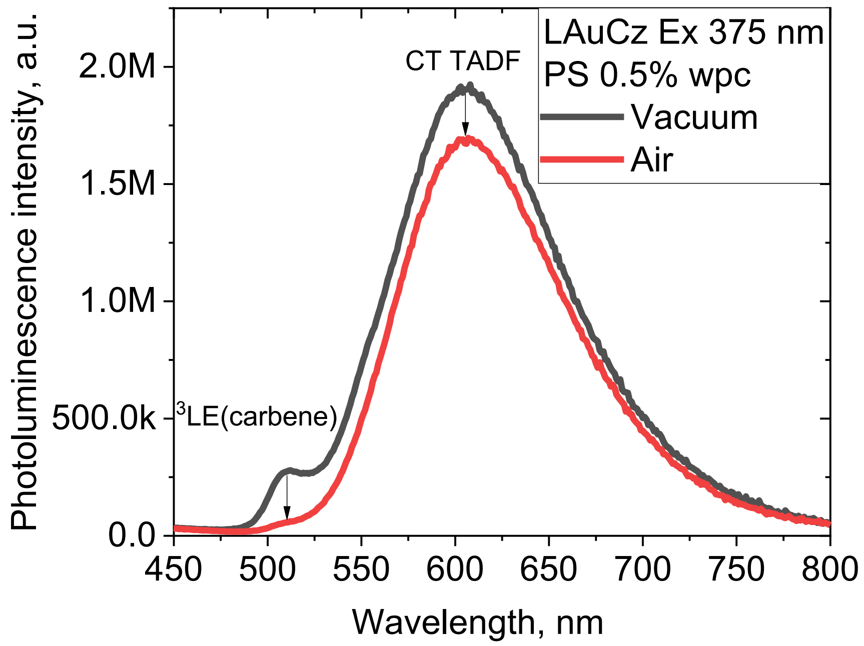

c)

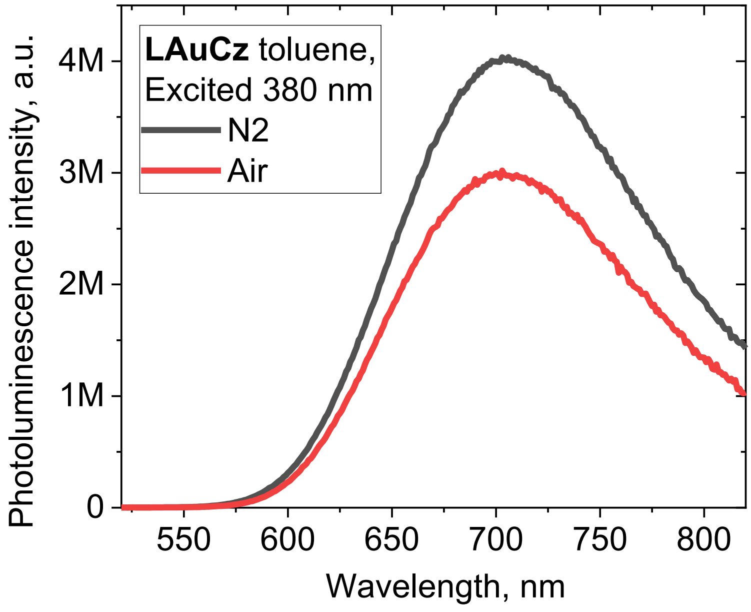

Figure S25. Further experiments corroborating TADF assignment from first singlet CT state for **LAuCz** complex in 0.5 wt.% PS thin film: (a) prompt and delayed PL profiles after 50 and 500 ns delay (top); b) oxygen quenching experiment demonstrating quenching of the ^{3}LE(carbene) phosphorescence and minor quenching of the CT TADF at 296 K (middle); c) oxygen quenching experiment demonstrating quenching of the CT component in toluene (bottom).

Optimised coordinates for LCuCl, LAuCl and LAuCz:

53

LCuCl s0 optimized geometry

N	-1.09595800	0.25610100	-0.01177000
N	1.09520600	0.25878700	-0.01195400
C	-0.71441600	6.20694300	-0.59721700
N	-1.43786700	2.65347600	-0.23428300
N	1.43122200	2.65697200	-0.23453900
C	-2.43155300	-0.25265100	0.03671600
C	-0.71379200	1.58694700	-0.12507100
C	-2.72250100	-1.42045100	-0.64888900
C	0.70976600	1.58868300	-0.12520400
C	0.00060500	-0.54499200	0.07000600
C	2.43203800	-0.24670000	0.03652000
C	-0.71812500	3.80757900	-0.35140300
C	0.70865200	3.80931200	-0.35153700
C	-3.39029400	0.38996200	0.80877500
C	1.39545500	5.03717700	-0.47732800
C	-3.98043100	-2.00487600	-0.52336400
C	3.38923600	0.39822100	0.80858200
C	-1.40792300	5.03377900	-0.47706000
C	0.69909200	6.20865500	-0.59735500
C	2.72578600	-1.41383100	-0.64901300
C	4.66313200	-0.14810500	0.91168400
C	-4.66292500	-0.15933500	0.91175100
C	3.98504600	-1.99536500	-0.52329500
C	4.93703400	-1.34869700	0.25612900
C	-4.93399600	-1.36047000	0.25602500
C	5.72726400	0.54151700	1.71760400
C	4.27460000	-3.29382100	-1.21892000
H	-1.24878200	7.14315800	-0.69437000

H	-1.96853400	-1.89121300	-1.27126500
H	-3.14159300	1.30323700	1.33425800
H	2.47740600	5.00862900	-0.47685700
H	3.13839300	1.31095400	1.33398700
H	-2.48980100	5.00261500	-0.47638400
H	1.23117200	7.14616100	-0.69461000
H	1.97299100	-1.88637300	-1.27146200
H	5.92171500	-1.79385100	0.36272800
H	-5.91767200	-1.80787200	0.36245500
H	6.35726200	-0.18109000	2.23688200
H	6.37542100	1.13707800	1.07079000
H	5.29057300	1.21265100	2.45692200
H	5.21522900	-3.72300500	-0.87535500
H	3.47307500	-4.01166700	-1.03288500
H	4.34282700	-3.15055500	-2.29929100
C	-5.72868000	0.52778000	1.71766800
H	-6.37615600	1.12454000	1.07127800
H	-6.35903900	-0.19641000	2.23427900
H	-5.29350700	1.19742400	2.45923800
C	-4.26704700	-3.30387000	-1.21920600
H	-3.46400800	-4.02002200	-1.03318600
H	-5.20676800	-3.73516400	-0.87579200
H	-4.33548000	-3.16060900	-2.29956300
Cu	0.00319100	-2.45624800	0.14559900
Cl	0.00637600	-4.55500800	-0.01623200

73

LAuCz s0 optimized geometry

N	-1.85601900	1.08650500	0.06236700
N	-1.78557200	-1.10904300	0.09089600
C	-7.81134600	0.50015200	0.33675800
N	-4.26972500	1.34439200	0.15823200
N	-4.17465400	-1.52416400	0.19187700

C	-1.43170500	2.45304500	0.03057200
C	-3.17619200	0.65572600	0.11503400
C	-0.42961300	2.87511200	0.89583700
C	-3.12993200	-0.76483000	0.13240600
C	-1.01623700	0.01363400	0.02982100
C	-1.27099700	-2.44512900	0.10662200
C	-5.40374700	0.58527800	0.22158500
C	-5.35661300	-0.84088500	0.23763800
C	-2.02391600	3.32260100	-0.86817100
C	-6.56491600	-1.56851800	0.30367000
C	0.01483500	4.19047500	0.84406800
C	-1.74771200	-3.36319200	-0.81907200
C	-6.65741100	1.23295600	0.27281500
C	-7.76468500	-0.91204900	0.35213600
N	2.94116400	0.05060500	-0.24264400
C	-0.30761800	-2.78910500	1.03960700
C	-1.23873600	-4.65544200	-0.82385000
C	-1.60145700	4.64979700	-0.92093200
C	0.22433200	-4.07838300	1.03990500
C	-0.25172800	-4.98958600	0.10428900
C	-0.58155700	5.05917100	-0.07003400
C	-1.74585700	-5.67893800	-1.79951000
C	3.77384000	1.14333800	-0.11868400
C	3.42859400	2.49491300	-0.05746400
C	4.44460800	3.42523400	0.07645400
C	5.78987600	3.03275300	0.14456300
C	6.13494400	1.69318800	0.07542000
C	5.12768400	0.73787000	-0.05750600
C	1.29854600	-4.45218600	2.02009200
C	3.75058000	-1.06605700	-0.27015200
C	5.11266400	-0.70135400	-0.15624000
C	6.09927200	-1.68687700	-0.15673000

C	5.72599500	-3.01597500	-0.27042100
C	4.37303000	-3.36721200	-0.39103800
C	3.37688200	-2.40568100	-0.39572100
H	-8.76911500	1.00266100	0.37698400
H	0.00489200	2.17623700	1.60162000
H	-2.80496600	2.96790900	-1.52956000
H	-6.49938500	-2.64876900	0.31620800
H	-2.50776000	-3.06655000	-1.53165400
H	-6.66334900	2.31520200	0.26160200
H	-8.68726800	-1.47563200	0.40384400
H	0.03219800	-2.05393900	1.76051300
H	0.15638200	-5.99591900	0.09717100
H	-0.23564200	6.08733600	-0.11971700
H	-0.92467200	-6.25909700	-2.22158200
H	-2.42291600	-6.37999600	-1.30671400
H	-2.29046300	-5.20858300	-2.61763500
H	2.38805300	2.79723800	-0.12909400
H	4.19844900	4.48032300	0.12071900
H	6.56117900	3.78573700	0.24721800
H	7.17521600	1.39066200	0.12355400
H	1.47066300	-5.52813900	2.02205400
H	2.23930500	-3.95759300	1.76512300
H	1.03116200	-4.14438200	3.03189200
H	7.14561700	-1.41586900	-0.06884800
H	6.48125300	-3.79186800	-0.27283700
H	4.10290900	-4.41260500	-0.49038300
H	2.33033100	-2.67549300	-0.50586200
C	-2.24291800	5.60909400	-1.88282900
H	-3.25115600	5.87019400	-1.55467600
H	-1.66643500	6.53020800	-1.96099000
H	-2.32654600	5.17164200	-2.87846100
C	1.10766000	4.67003100	1.75676900

H	1.86132400	5.22896500	1.19921300
H	0.70902300	5.33389800	2.52668000
H	1.60730100	3.83543700	2.24798500
Au	0.95439700	0.04715100	-0.11652800

73

LAuCz s1 optimized geometry

N	1.81647900	1.08996700	-0.15915900
N	1.81685000	-1.08974300	-0.15920500
C	7.81705800	0.69976500	-0.01135200
N	4.22630400	1.45894100	-0.07805300
N	4.22681100	-1.45787800	-0.07837500
C	1.36788400	2.43871100	-0.18620700
C	3.14957000	0.69953000	-0.09808000
C	0.39503400	2.82083900	-1.10151900
C	3.14981400	-0.69884000	-0.09822200
C	0.99304000	-0.00003300	-0.18021500
C	1.36877600	-2.43866800	-0.18597500
C	5.38656800	0.71886700	-0.05717500
C	5.38681700	-0.71740400	-0.05729800
C	1.91265000	3.35866200	0.69682900
C	6.61975900	-1.38823600	-0.03445600
C	-0.06988600	4.13070500	-1.11896000
C	1.91415400	-3.35820700	0.69709500
C	6.61928200	1.39011400	-0.03416300
C	7.81729900	-0.69748300	-0.01146300
N	-3.02533000	-0.00032700	0.20056100
C	0.39579800	-2.82139700	-1.10092700
C	1.47074100	-4.67727300	0.68413900
C	1.46869600	4.67753000	0.68344900
C	-0.06855600	-4.13145800	-1.11794900
C	0.47459700	-5.04325800	-0.21687700
C	0.47265400	5.04291400	-0.21795700

C	2.07532000	-5.68598300	1.61955600
C	-3.95118700	-0.00056600	-0.81113700
C	-3.70329700	-0.00100100	-2.18458900
C	-4.79880800	-0.00118900	-3.03787900
C	-6.09827900	-0.00096200	-2.53336200
C	-6.34666500	-0.00053300	-1.15229500
C	-5.27158000	-0.00033800	-0.29319700
C	-1.10589400	-4.55805100	-2.11892500
C	-3.71151500	0.00004500	1.37997900
C	-5.11505700	0.00007400	1.15576800
C	-5.97965500	0.00044400	2.22442200
C	-5.43981800	0.00079000	3.52160500
C	-4.06218400	0.00076000	3.73803800
C	-3.17475200	0.00039000	2.67203000
H	8.75349300	1.24323900	0.00548300
H	0.01736500	2.09170600	-1.80981100
H	2.68983500	3.04275000	1.38071500
H	6.59230700	-2.47103400	-0.03804300
H	2.69134500	-3.04182000	1.38075200
H	6.59146200	2.47290400	-0.03744200
H	8.75392300	-1.24063800	0.00519700
H	0.01755600	-2.09257200	-1.80922400
H	0.11752700	-6.06906600	-0.22244600
H	0.11524600	6.06859700	-0.22392100
H	1.45922200	-6.58295100	1.68373500
H	3.06751400	-5.98371100	1.27406600
H	2.19190000	-5.27428100	2.62270500
H	-2.68603000	-0.00118100	-2.55399300
H	-4.64390400	-0.00152500	-4.10802600
H	-6.93364900	-0.00112400	-3.22064500
H	-7.36391100	-0.00036800	-0.78124700
H	-1.64641000	-5.43968400	-1.77378500

H	-1.82846500	-3.76127100	-2.30166600
H	-0.64310500	-4.80804800	-3.07632000
H	-7.05289100	0.00048300	2.08129100
H	-6.10935200	0.00109500	4.37124500
H	-3.68312500	0.00104100	4.75054000
H	-2.10235300	0.00038300	2.81407300
C	2.07249900	5.68662000	1.61896100
H	3.06655500	5.98135100	1.27629400
H	1.45824200	6.58510300	1.67939900
H	2.18471500	5.27656700	2.62329600
C	-1.10723200	4.55644500	-2.12029300
H	-1.64356400	5.44197700	-1.77866100
H	-0.64513800	4.79961900	-3.07977400
H	-1.83337500	3.76169400	-2.29772800
Au	-0.99142500	-0.00025200	-0.00740300

73

LAuCz t1 optimized geometry

N	-1.82800500	1.08963400	0.14475900
N	-1.82566500	-1.09004400	0.14683300
C	-7.82904700	0.69170100	0.05667500
N	-4.23965400	1.45579400	0.08442400
N	-4.23631900	-1.46183800	0.08718200
C	-1.37188500	2.43585300	0.14776100
C	-3.16151100	0.69742700	0.09531300
C	-0.36545800	2.81926800	1.03005000
C	-3.15996500	-0.70092000	0.09659700
C	-1.00448700	0.00068500	0.15979600
C	-1.36654100	-2.43524000	0.15240900
C	-5.39847300	0.71404700	0.07653800
C	-5.39683800	-0.72278800	0.07785400
C	-1.93271800	3.35087800	-0.72727000
C	-6.62936300	-1.39527300	0.06852900

C	0.11584200	4.12192100	1.01334200
C	-1.92325900	-3.35245900	-0.72335800
C	-6.63252200	1.38370900	0.06620600
C	-7.82745200	-0.70599800	0.05782400
N	3.00914900	0.00127000	-0.18929400
C	-0.36096100	-2.81515500	1.03672500
C	-1.45796000	-4.66590600	-0.74146700
C	-1.47112300	4.66588100	-0.74703900
C	0.12435200	-4.11661400	1.02157900
C	-0.42975400	-5.02680500	0.12246900
C	-0.44297200	5.03035300	0.11497400
C	-2.07083500	-5.66712500	-1.67921700
C	3.81752500	1.10416800	-0.22360000
C	3.41965300	2.44353900	-0.20072700
C	4.41621700	3.40885800	-0.23861900
C	5.76194000	3.04722900	-0.29943700
C	6.15914400	1.70103900	-0.32511300
C	5.18529400	0.72937800	-0.28713500
C	1.20303600	-4.54209300	1.97965200
C	3.81609700	-1.10219300	-0.23140700
C	5.18439800	-0.72869300	-0.29229200
C	6.15696100	-1.70132900	-0.33710900
C	5.75800500	-3.04715900	-0.32084800
C	4.41178400	-3.40754800	-0.26260600
C	3.41650300	-2.44127600	-0.21803400
H	-8.76645000	1.23368600	0.04938300
H	0.02348000	2.09771400	1.73993300
H	-2.73408900	3.03637900	-1.38325900
H	-6.60063300	-2.47802000	0.07267300
H	-2.72381000	-3.04033100	-1.38147900
H	-6.60626100	2.46652500	0.06845400
H	-8.76362100	-1.25012400	0.05143000

H	0.02510900	-2.09182700	1.74637000
H	-0.05767100	-6.04725900	0.10499900
H	-0.07466500	6.05216600	0.09709700
H	-1.50163700	-6.59652100	-1.69037200
H	-3.09384300	-5.90008300	-1.37767100
H	-2.11445800	-5.27795300	-2.69736900
H	2.36795700	2.70217300	-0.15840800
H	4.14620600	4.45622800	-0.22398400
H	6.51764700	3.82086300	-0.32859200
H	7.20993300	1.44390100	-0.37302800
H	1.91041800	-5.22219900	1.50081800
H	1.75640000	-3.68159800	2.35858400
H	0.77927200	-5.06745400	2.83816500
H	7.20809000	-1.44525300	-0.38320300
H	6.51273100	-3.82153100	-0.35538900
H	4.14043400	-4.45464100	-0.25531300
H	2.36448000	-2.69886900	-0.17754200
C	-2.08643800	5.66314100	-1.68742800
H	-3.12715800	5.85385100	-1.41917800
H	-1.55019300	6.61161600	-1.66190300
H	-2.07973200	5.29249300	-2.71356600
C	1.19574400	4.55154800	1.96820600
H	1.91874400	5.20653500	1.47754700
H	0.77637400	5.10612600	2.81028400
H	1.73098700	3.69083200	2.37169200
Au	0.97863000	0.00155100	-0.01801600

53

LAuCl s0 optimized geometry

N	0.64071800	1.09810400	-0.07111200
N	0.64067800	-1.09833200	-0.07097500
C	6.60307900	0.70649700	-0.47762900
N	3.04070100	1.43467000	-0.22457300

N	3.04066100	-1.43498400	-0.22436600
C	0.17702100	2.45198200	-0.03888800
C	1.97218000	0.71024700	-0.14759400
C	-0.84080500	2.84021700	-0.89162500
C	1.97216600	-0.71051700	-0.14751300
C	-0.16419400	-0.00009200	-0.00990900
C	0.17683500	-2.45216500	-0.03887100
C	4.19733600	0.71331700	-0.30614500
C	4.19731800	-0.71368400	-0.30602700
C	0.75056900	3.34067000	0.86068200
C	5.42746700	-1.40188400	-0.39362700
C	-1.33682800	4.14094100	-0.82575200
C	0.75049900	-3.34111500	0.86036400
C	5.42746300	1.40154400	-0.39388500
C	6.60306600	-0.70681500	-0.47749000
C	-0.84133700	-2.84008000	-0.89135200
C	0.28616000	-4.64855800	0.91901100
C	0.28651000	4.64820200	0.91936900
C	-1.33764700	-4.14069100	-0.82548400
C	-0.76187700	-5.02501900	0.07858800
C	-0.76112500	5.02502400	0.07860200
C	0.90274600	-5.64122700	1.86318300
C	-2.48137800	-4.55154700	-1.70723900
H	7.54250000	1.23974200	-0.54566300
H	-1.25762600	2.12826100	-1.59447200
H	1.54921300	3.00983900	1.51319300
H	5.39756700	-2.48378900	-0.39345900
H	1.54940600	-3.01055700	1.51268600
H	5.39745700	2.48344600	-0.39391200
H	7.54247400	-1.24010400	-0.54539700
H	-1.25817300	-2.12799900	-1.59405800
H	-1.14280500	-6.04030500	0.13618700

H	-1.14176200	6.04042500	0.13612100
H	0.14245200	-6.28128400	2.31171000
H	1.60771500	-6.28852900	1.33682900
H	1.44624800	-5.13978900	2.66358100
H	-2.72589200	-5.60418300	-1.56914400
H	-3.37030000	-3.96012500	-1.47974900
H	-2.24315900	-4.39075500	-2.75998900
C	0.90298000	5.64060600	1.86389600
H	1.60808900	6.28799200	1.33783500
H	0.14263500	6.28059800	2.31243100
H	1.44629100	5.13893600	2.66427700
C	-2.48036000	4.55215700	-1.70759000
H	-3.37066300	3.96390700	-1.47724300
H	-2.72187800	5.60588000	-1.57254200
H	-2.24378100	4.38729700	-2.76006700
Cl	-4.38148600	0.00040900	0.32372500
Au	-2.13566000	0.00012500	0.15509200

Supplementary References.

[1] Programs CrysAlisPro, Oxford Diffraction Ltd. Abingdon, UK, 2010.

[2] G. Sheldrick, Crystal structure refinement with SHELXL. *Acta Cryst. C* **2015,** *71* (1), 3-8.

[3] O. V. Dolomanov, L. J. Bourhis, R. J. Gildea, J. A. K. Howard, H. Puschmann, OLEX2: a complete structure solution, refinement and analysis program. *J. Appl. Cryst.* **2009,** *42* (2), 339.

[4] Gritzner, G.; Kůta, J. Recommendations on reporting electrodepotentials in nonaqueous solvents: IUPC commission on electro-chemistry. *Electrochim. Acta*, 1984, 29, 869−873.

[5] M. A. Albota, C. Xu, W. W. Webb, *Appl. Opt.*, 1998, **37** (31), 7352.

[6] C. Xu, W. W. Webb, *J. Opt. Soc. Am. B*, 1996, **13** (3), 481.

[7] J. Daniel, website link: https://github.com/LAGONteam/PyTwoPhotonExcitedFluorescence.

[8] R.J. Marsh et al. / Chemical Physics Letters 366 (2002) 398–405
[9] Teodora Scheul, Ciro D'Amico, Irène Wang, and Jean-Claude Vial, "Two-photon excitation and stimulated emission depletion by a single wavelength," Opt. Express 19, 18036-18048 (2011)
[10] F. Furche and D. Rappoport, Density functional methods for excited states: equilibrium structure and electronic spectra. In Computational Photochemistry; M. Olivuccim, Ed.; Elsevier: Amsterdam, 2005; pp. 93–128.
[11] G. M. J. Peach and D. J. Tozer, *J. Phys. Chem. A,* 2012, **116**, 9783–9789.
[12] H. S. Yu, X. He, S. L. Li and D. G. Truhlar, *Chem. Sci*., 2016, **7**, 5032–5051.
[13] F. Weigend, M. Häser, H. Patzelt and R. Ahlrichs, *Chem. Phys. Lett.,* 1998, **294**, 143–152.
[14] F. Weigend and R. Ahlrichs, *Phys. Chem. Chem. Phys.,* 2005, **7**, 3297–3305.
[15] D. Andrae, U. Haeussermann, M. Dolg, H. Stoll and H. Preuss, *Theor. Chim. Acta*, 1990, **77**, 123–141.
[16] F. Chotard, A. S. Romanov, D. L. Hughes, M. Linnolahti and M. Bochmann, *Eur. J. Inorg. Chem.,* 2019, 4234–4240.
[17] A. S. Romanov, S. T. E. Jones, Q. Gu, P. J. Conaghan, B. H. Drummond, J. Feng, F. Chotard, L. Buizza, M. Foley, M. Linnolahti, D. Credgington and M. Bochmann, *Chem. Sci*., 2020, **11**, 435–446.
[18] T. Lu and F. J. Chen, *Comput. Chem*., 2012, **33**, 580–592.
[19] Gaussian 16, Revision A.03, M.J. Frisch, G.W. Trucks, H.B. Schlegel, G.E. Scuseria, M.A. Robb, J.R. Cheeseman, G. Scalmani, V. Barone, G.A. Petersson, H. Nakatsuji, X. Li, M. Caricato, A.V. Marenich, J. Bloino, B.G. Janesko, R. Gomperts, B. Mennucci, H.P. Hratchian, J.V. Ortiz, A.F. Izmaylov, J.L. Sonnenberg, D. Williams-Young, F. Ding, F. Lipparini, F. Egidi, J. Goings, B. Peng, A. Petrone, T. Henderson, D. Ranasinghe, V.G. Zakrzewski, J. Gao, N. Rega, G. Zheng, W. Liang, M. Hada, M. Ehara, K. Toyota, R. Fukuda, J. Hasegawa, M. Ishida, T. Nakajima, Y. Honda, O. Kitao, H. Nakai, T. Vreven, K. Throssell, J.A. Montgomery, Jr., J.E. Peralta, F. Ogliaro, M.J. Bearpark, J.J. Heyd, E.N. Brothers, K.N. Kudin, V.N. Staroverov, T.A. Keith, R. Kobayashi, J. Normand, K. Raghavachari, A P. Rendell, J.C. Burant, S.S. Iyengar, J. Tomasi, M. Cossi, J.M. Millam, M. Klene, C. Adamo, R. Cammi, J.W. Ochterski, R.L. Martin, K. Morokuma, O. Farkas, J.B. Foresman and D.J. Fox, Gaussian, Inc., Wallingford CT, 2016.
[20] Neese, F. *Software update: the ORCA program system -- Version 5.0 Wiley Interdiscip. Rev.: Comput. Mol. Sci.*, **2022**, *12*, *1*, e1606.
[21] Tsuchiya, S. Diesing, F. Bencheikh, Y. Wada, P. L. dos Santos, H. Kaji, E. Zysman-Colman, I. D. W. Samuel, C. Adachi, *J. Phys. Chem. A* **2021**, 125, 36, 8074-8089
