# Supplementary material for: Enhanced third-order optical nonlinearity in a dipolar carbene-metal-amide material with two-photon excited delayed fluorescence
Source: Commun Chem. 2026 Feb 17;9:135. doi: 10.1038/s42004-026-01928-5 (PMC13022332; doi:10.1038/s42004-026-01928-5)
Supplement: Supplementary file 3 — Description of Additional Supplementary Files [file 42004_2026_1928_MOESM3_ESM.pdf]

Description of Additional Supplementary Files:

File name: Supplementary Data

Description: Optimised coordinates
